# Cultivation and genomic characterization of novel and ubiquitous marine nitrite-oxidizing bacteria from the *Nitrospirales*

**DOI:** 10.1038/s41396-023-01518-6

**Published:** 2023-09-25

**Authors:** Anna J. Mueller, Anne Daebeler, Craig W. Herbold, Rasmus H. Kirkegaard, Holger Daims

**Affiliations:** 1 University of Vienna, Division of Microbial Ecology, Centre for Microbiology and Environmental Systems Science, Djerassiplatz 1, 1030 Vienna, Austria; 2 Doctoral School in Microbiology and Environmental Science, University of Vienna, Djerassiplatz 1, 1030 Vienna, Austria; 3 Department of Soil Biology and Biogeochemistry, Biology Centre CAS, Na Sádkách 7, 370 05 Budweis, Czech Republic; 4 School of Biological Sciences, University of Canterbury, Christchurch, 8041 New Zealand; 5 Joint Microbiome Facility of the Medical University of Vienna and the University of Vienna, Vienna, Austria; 6 The Comammox Research Platform, University of Vienna, Vienna, Austria

**Keywords:** Marine microbiology, Bacterial genomics

## Abstract

*Nitrospirales*, including the genus *Nitrospira*, are environmentally widespread chemolithoautotrophic nitrite-oxidizing bacteria. These mostly uncultured microorganisms gain energy through nitrite oxidation, fix CO_2_, and thus play vital roles in nitrogen and carbon cycling. Over the last decade, our understanding of their physiology has advanced through several new discoveries, such as alternative energy metabolisms and complete ammonia oxidizers (comammox *Nitrospira*). These findings mainly resulted from studies of terrestrial species, whereas less attention has been given to marine *Nitrospirales*. In this study, we cultured three new marine *Nitrospirales* enrichments and one isolate. Three of these four NOB represent new *Nitrospira* species while the fourth represents a novel genus. This fourth organism, tentatively named “*Ca*. Nitronereus thalassa”, represents the first cultured member of a *Nitrospirales* lineage that encompasses both free-living and sponge-associated nitrite oxidizers, is highly abundant in the environment, and shows distinct habitat distribution patterns compared to the marine *Nitrospira* species. Partially explaining this, “*Ca*. Nitronereus thalassa” harbors a unique combination of genes involved in carbon fixation and respiration, suggesting differential adaptations to fluctuating oxygen concentrations. Furthermore, “*Ca*. Nitronereus thalassa” appears to have a more narrow substrate range compared to many other marine nitrite oxidizers, as it lacks the genomic potential to utilize formate, cyanate, and urea. Lastly, we show that the presumed marine *Nitrospirales* lineages are not restricted to oceanic and saline environments, as previously assumed.

## Introduction

Nitrate (NO_3_^-^) is the most abundant bioavailable nitrogen species in the oceans, and nitrogen availability is often a key factor limiting the growth of marine microorganisms and phytoplankton [1]. The second step of nitrification, nitrite (NO_2_^-^) oxidation to nitrate, is the main biological nitrate-forming process in the environment. Aerobic chemolithoautotrophic nitrite-oxidizing bacteria (NOB) are the main organisms conducting this step in the oceans and thus play a crucial role in the marine nitrogen cycle. The dominant marine NOB from the *Nitrospinales* are mostly uncultured, but members of the genera *Nitrospira*, *Nitrococcus*, *Nitrobacter*, and *Nitrotoga* are also known to perform nitrite oxidation in marine systems [2]. Especially *Nitrospira* are often detected in marine habitats, although they are generally much less abundant than *Nitrospinales* in the ocean water column [3]. However, *Nitrospira* have been found to be both prevalent and abundant in marine sponges, where *Nitrospinales* are only found sporadically [4–6]. *Nitrospira* also seem to be abundant in marine sediments, some surface water regions, the hadal zone, and hydrothermal vents [3, 7, 8].

Since the description of the first *Nitrospira* species in 1986, the marine strain *Nitrospira marina* Nb-295 [9], members of this genus have been discovered in a large variety of natural and engineered ecosystems. *Nitrospira* is a diverse NOB genus and contains at least six monophyletic sub-lineages designated as *Nitrospira* lineages I to VI [10–12]. Of these, mainly members of lineage IV (which contains the type strain *N. marina* Nb-295) have been found in the oceans and other saline aquatic systems [9, 13–15].

In contrast to the ocean, *Nitrospira* are the dominant NOB in many terrestrial ecosystems, including wastewater and drinking water treatment plants, groundwater, lakes, hot springs, and soils [2]. Thus, it is not surprising that research on *Nitrospira* to date has focused more on terrestrial rather than marine systems [2]. The broad distribution of *Nitrospira* in many different habitats likely reflects their adaptation to low nitrite concentrations, as found in most environments [16], and their ability to also use metabolisms other than nitrite oxidation for energy generation. Indeed, past studies found that *Nitrospira* members can gain energy from oxidizing hydrogen at elevated or even at atmospheric concentrations [17, 18] and from oxidizing formate with oxygen or nitrate as the terminal electron acceptor [19, 20]. Nitrite-oxidizing *Nitrospira* can also indirectly utilize organic nitrogen compounds, such as urea and cyanate, as energy sources *via* reciprocal feeding interactions with ammonia-oxidizing microorganisms [19, 21]. Furthermore, complete ammonia-oxidizing (comammox) *Nitrospira* were recently discovered [22, 23], breaking the paradigm that the two nitrification steps are catalyzed by different organisms. However, due to the recalcitrance to cultivation of most environmental *Nitrospira*, our knowledge of their metabolic flexibility and ecological niche partitioning remains severely limited. For example, only two marine *Nitrospira* strains have been isolated [9, 14].

In this study, we explored the nitrite-oxidizing activity, phylogeny, environmental abundance, and genomic potential of marine *Nitrospirales* by leveraging three new enrichments and one new isolate, which nearly doubles the number of cultured marine NOB species from this group. One of these enrichments contains the first cultured representative of a hitherto uncultured lineage. This organism is also a close free-living relative of the marine sponge-associated NOB.

## Materials and methods

### Sampling and setup of nitrite-oxidizing enrichment cultures

Water column samples were taken at approximately 1 m depth at three different coastal locations: Burrard Inlet, Vancouver, Canada (49°16’22.1”N 123°11’32.5”W) and Elba, Italy (42°43’48.1”N 10°09’23.2”E) (both in November 2016); and Slettestrand, Denmark (57°09’26.3”N 9°21’55.5”E) in September 2017. A fourth sample was taken from coastal surface sediments: Maine, USA (43°6’31”N 70°39’56”W) in August 2016.

Nitrite-oxidizing enrichment cultures were started by directly adding 0.25 to 0.5 mM NO_2_^-^ to the sampled seawater for the Vancouver and Denmark samples. The Maine enrichment was started by adding a spatula of sediment to a marine minimal medium prepared with seawater from the North Sea [24] and 0.5 mM NO_2_^-^. Microorganisms in the sample from Elba were first concentrated by filtration of 100 ml seawater on a sterile 0.22 μm polycarbonate filter (Merck Millipore), which was then used to inoculate 25 ml of marine mineral medium prepared with red sea salt (Red Sea Aquaristic) supplemented with vitamins and 0.5 mM NO_2_^-^ [25]. The cultures were routinely checked for nitrite consumption and nitrate formation with test stripes (Macherey-Nagel). All enrichments, except the Denmark enrichment (see below), were regularly transferred (dilution factor 1:10) to fresh medium and were diluted more strongly at least once (dilution factor between 1:10^2^ and 1:10^7^). The Vancouver enrichment was routinely grown on 31 ppt marine mineral medium prepared with red sea salt (Red Sea Aquaristic) supplemented with vitamins [25] and 0.5 to 1 mM NO_2_^-^. The Maine enrichment was further grown in the same medium as mentioned above with 0.5 to 1 mM NO_2_^-^. The Elba enrichment was initially grown on red sea salt based minimal medium (see above). After two transfers, however, nitrite oxidation ceased and this enrichment was further successfully cultured in the North Seawater-based marine mineral medium. The Elba enrichment was routinely supplemented with 0.25 mM NO_2_^-^ after nitrite consumption, since the addition of 0.5 mM NO_2_^-^ had caused a decline of the nitrite-oxidizing activity, suggesting that this culture was more sensitive to higher nitrite concentrations than the other enrichments. All enrichments were kept at either room temperature (~22 °C, Denmark enrichment) or 28 °C (Elba, Maine, Vancouver enrichments) in the dark without agitation.

### Isolation and cultivation of the Denmark strain

After 9 months of enrichment and consumption of 7.25 mM nitrite, the Denmark culture was subjected to a previously published [25] random cell sorting and activity screening approach to purify the NOB. Briefly, cells were randomly sorted, by using a MoFlo Astrios Flow Cytometer (Beckman Colter) equipped with a 70 µm air jet in nozzle and pressure of 60 psi, into one 96-well plastic microtiter plate (Corning) that had been pre-filled with 200 µl of the red sea salt based marine mineral medium supplemented with vitamins, 0.25 mM NO_2_^-^, and 0.1 mM pyruvate for oxidative stress alleviation. The microtiter plate was incubated at room temperature in plastic bags (to minimize evaporation) in the dark and routinely checked for nitrite consumption with the Griess Assay [26]. Wells showing nitrite-consuming activity were sub-cultured and gradually scaled up, after each round of NO_2_^-^ depletion, by doubling the total medium volume up to 5 ml in larger microtiter plates and cell culture flasks (CELLSTAR, Greiner). Subsequently, the cultures were transferred into 50 ml fresh medium in 100 ml Schott bottles. From this point on, the cultures were routinely grown as described above in red sea salt based marine mineral medium supplemented with vitamins and 0.5 to 1 mM NO_2_^-^ at 28 °C in the dark. NOB obtained through cell sorting were preliminarily identified as *Nitrospira* members through direct Sanger sequencing of the 16S rRNA gene, which had been amplified by PCR with the universal bacterial 16S rRNA-targeted primers 27F and 1492R [27]. Culture aliquots were streaked out on Marine broth 2216 agar (BD Difco) to confirm the absence of contaminating heterotrophic bacteria.

### Nitrite-oxidizing activity analyses

Aliquots of 50 ml of nitrite-depleted enrichment cultures were centrifuged at 4500 × *g* for 20 min at 28 °C. The cultures were then washed by resuspending the cells in 2.5 ml of their respective medium. After a second centrifugation step, the cells were resuspended in 500 µl of medium. Aliquots of 100 µl of the concentrated cells were then each inoculated in quadruplicates into 50 ml of their respective medium amended with 1 mM nitrite (Maine, Vancouver, Denmark cultures) or 0.25 mM nitrite (Elba enrichment) and incubated at 28 °C without agitation in the dark. During the incubation period of up to 14 days, aliquots of the cultures were regularly sampled by centrifugation (14000 rpm for 10 min at 4 °C) and the supernatant was stored at −20 °C prior to measuring the NO_2_^-^ and NO_3_^-^ concentrations in the supernatant colorimetrically with the Griess assay as described previously [22, 26].

### Phylogenetic analyses

The genomes and metagenome assembled genomes (MAGs) (Table S1) were obtained from co-assemblies of Illumina and Nanopore reads (Supplementary Materials and Methods). The genomes were annotated in the MicroScope platform (v3.16.0) [28].

All available *Nitrospirales* assemblies were downloaded from the NCBI database (April 2022). Their quality and taxonomic affiliations were assessed with CheckM (v1.2.0) [29] and the Genome Taxonomy Database toolkit (GTDB-tk) (v2.1.0) [30], respectively. For all subsequent analyses, a *Nitrospirales* dataset was selected based on CheckM quality cutoffs (≥80% genome completeness and <10% contamination) and the GDTB-tk taxonomy assignment to “*Nitrospirales*” (Table S2). Based on this dataset and a concatenated alignment of conserved marker proteins determined by GDTB-tk, a phylogenetic tree was calculated using iQTree (v2.1.2) [31] with the automatic model finder (selected model:JTT + F + R7) [32] and 1000 ultra-fast bootstrap iterations [33].

For a phylogenetic analysis based on 16S rRNA genes, *Nitrospirales* sequences were extracted from the Silva RefNR database release r138.1 [34] with the following settings: taxonomy *Nitrospirales*, sequence length >1399 nucleotides (nt), sequence quality >90, pintail quality >90. The resulting dataset was combined with 16S rRNA gene sequences (length >1399 nt) extracted from the aforementioned *Nitrospirales* genomic dataset. SINA (v1.2.11) [35] was used for the alignment of the 16S rRNA gene sequences, and a phylogenetic tree was calculated using iQTree (v2.1.2) with the automatic model finder (selected model: TIM3e + R5), 1000 ultra-fast bootstrap iterations, and the 16S rRNA gene sequence of *Leptospirillum ferrooxidans* as outgroup. The resulting tree was then used to select the 16S rRNA sequences of the lineage IV *Nitrospirales*, and a phylogenic tree of these sequences was calculated by the same approach as described above except for the selected model (TIM3e+I + G4) and the use of *N. moscoviensis* as the outgroup. Information on the sampling sites of the sequences was extracted from the Silva database or the associated publications. Phylogenetic trees were visualized in FigTree (v1.4.4) and edited in Adobe Illustrator.

Genome-wide average nucleotide identity (gANI) and average amino acid identity (AAI) values were calculated as described elsewhere [36, 37]. ANI and AAI were visualized as heatmaps using R (v3.6.1) with the tidyverse (v1.3.0) package.

### Distribution and abundance analysis of selected marine NOB

The global habitat distribution and abundance of selected marine *Nitrospirales*, *Nitrospinales*, and closely related organisms was assessed based on the frequencies of their 16S rRNA genes in published datasets by using the IMNGS platform [38]. The full-length 16S rRNA gene identities of the marine NOB cultured in this study and other relevant *Nitrospirales* (Table S3) were first compared against each other with BLASTn (v 2.11.0) (Fig. S1) to determine an appropriate sequence identity threshold for the actual analysis. This threshold was set at 97% nucleotide identity. The 16S rRNA sequence of *N. marina* Nb-295 was selected as the representative of the genus *Nitrospira* (lineage IVa), since the newly cultured Denmark, Maine and Vancouver *Nitrospira* share a very high (>99%) 16S rRNA identity (Fig. S1) with *N. marina* Nb-295.

The full-length 16S rRNA sequences were submitted to the IMNGS (https://www.imngs.org/) “Parallel Similarity” search against all database sequences (December 2021) with a similarity threshold of 97% and a minimum size of 200 nucleotides. The IMNGS classification was used to determine the environments which the matching sequences had been sourced from. If an environment could not be clearly deducted from the IMNGS classification, the sequence sampling site was looked up directly in the NCBI Sequence Read Archive. The environments were then assigned to one of the following categories: marine, marine host-associated, marine sediment, engineered, freshwater or freshwater sediment, terrestrial, terrestrial host-associated, or other (see Table S4). The data were analyzed and visualized using the R package tidyverse (v1.3.0).

## Results and discussion

### Cultivation and nitrite-oxidizing activity of marine NOB

Nitrite-oxidizing enrichment cultures were obtained from four distinct coastal marine sites: seawater samples from the Pacific Ocean (Vancouver, Canada), the North Sea (Slettestrand, Denmark), the Mediterranean Sea (Elba, Italy), and coastal surface sediments from the Atlantic Ocean (Maine, USA). The enrichment cultures were started by adding 0.25 to 0.5 mM NO_2_^-^ directly to the sampled seawater, or by adding sediment to natural seawater-based medium containing 0.5 mM NO_2_^-^. This approach resulted in detectable nitrite-oxidizing activity in all enrichments, except the Elba culture. Therefore, cells in the Elba seawater sample were pre-concentrated by filtration and then inoculated into marine mineral medium supplemented with vitamins and 0.5 mM NO_2_^-^. Actively nitrite-oxidizing enrichment cultures were regularly transferred into their respective marine mineral nitrite medium (see Materials and Methods for details).

After several rounds of nitrite consumption, the enrichments were subjected to random single-cell sorting by fluorescence-activated cell sorting (FACS) and activity screening in microtiter plates [25]. After two months of incubation after sorting, nitrite-oxidizing activity was observed only for the Denmark enrichment in two microtiter plate wells. These cultures were subsequently scaled up and identified as *Nitrospira* with identical 16S rRNA genes. Sub-cultivation on organic medium (see above) showed the absence of heterotrophs, and genome sequencing confirmed that these cultures were axenic. The Denmark isolate was obtained in nine months and with only one round of cell sorting. In comparison, the isolation of marine NOB by more traditional cultivation techniques took several years [14, 39]. For the remaining three enrichments, the lack of observed activity after cell sorting could have been caused by randomly sorting only non-NOB cells into the microtiter plates, by the metabolic state of the NOB, by failure of the NOB to cope as single cells with the conditions in the medium after sorting (e.g., failure to mitigate oxidative stress), or by other unidentified factors. Still, our results suggest that random single-cell sorting is a viable approach to relatively quickly isolate or highly enrich marine NOB. In a previous study, we had already used this method to obtain stable two-member co-cultures of two previously uncultured marine *Nitrospinales* strains [25]. Manual cell sorting with optical tweezers, or FACS-assisted sorting of microcolonies, also facilitated the isolation of non-marine *Nitrospira* strains [40–42]. To further improve their success rate, cell sorting-based isolation approaches may benefit from strain-specific media optimizations and repeated sorting rounds with enrichment samples from different growth stages to account for differences in optimal growth conditions and varying physiological states of the target NOB.

**Fig. 1:**
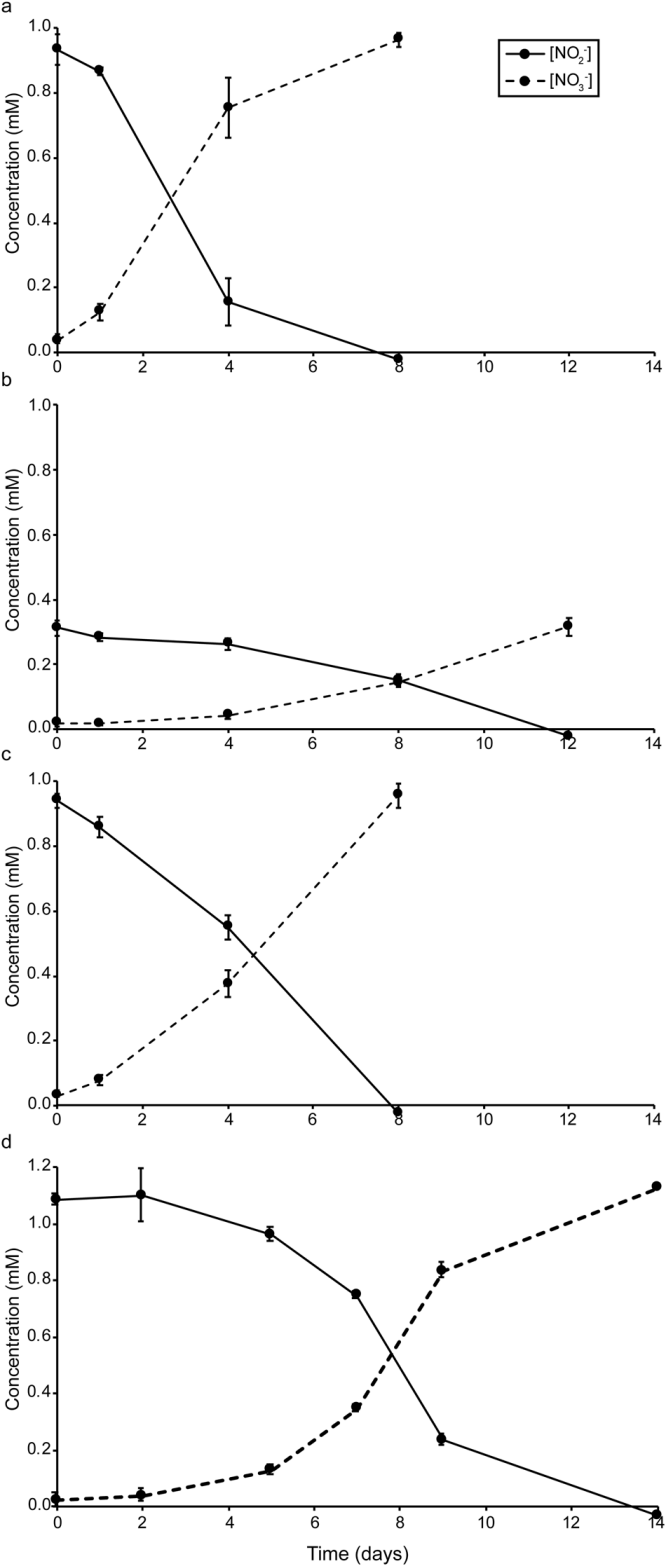
Nitrite-oxidizing activity of the newly cultured *Nitrospirales* members. Nitrite-oxidizing activity of **a** the Maine enrichment, **b** the Elba enrichment, **c** the Vancouver enrichment, and **d** the Denmark isolate during 8 to 14 days of incubation. The rates of nitrite consumption cannot be compared across cultures, because the total biomass and NOB densities differed. The full lines show nitrite and dashed lines nitrate concentrations. Values are means ± standard deviations (error bars) for biological quadruplicates.

Stoichiometric conversion of nitrite to nitrate was observed for the three enrichments from Vancouver, Maine, and Elba and for the Denmark isolate (Fig. 1). Nitrite concentrations above 0.25 mM caused the activity of the Elba enrichment to slow down noticeably, whereas the other two enrichments and the Denmark isolate were regularly replenished with 0.5 to 1 mM nitrite without any observed decrease in activity.

*Nitrospira* cells in the enrichments and the Denmark isolate were visualized by 16S rRNA-targeted fluorescence in situ hybridization (FISH) or catalyzed reporter deposition (CARD-FISH) (Supplementary Materials and Methods). All newly cultivated marine *Nitrospirales* displayed the typical spiral cell morphology of *Nitrospirales* (Fig. S2 a–h). Some of the cultures formed cell aggregates, a phenotype frequently observed for *Nitrospira* [5, 13, 14, 22, 40] although the tendency of these NOB to flocculate can vary based on the growth stage and conditions [43]. A considerable portion of non-*Nitrospirales* microbial cells was detected in the three enrichments (Fig. S2 a–f). Consistently, the metagenomic datasets of the enrichments contained a diverse range of putatively heterotrophic bacteria (Table S5). The CARD-FISH results also supported purity of the Denmark strain, as all general nucleic acid stain (DAPI) signals were congruent with *Nitrospira* specific CARD-FISH signals in this culture (Fig. S2 g).

### Phylogeny of the newly cultured marine NOB

High quality draft (96% completeness, <3% contamination) metagenome assembled genomes (MAGs) (Elba, Maine) and two closed genomes (Vancouver, Denmark) were obtained from the four newly cultured NOB. The genomes differ noticeably in size, between 4 and 4.8 Mbp (Table S1).

**Fig. 2:**
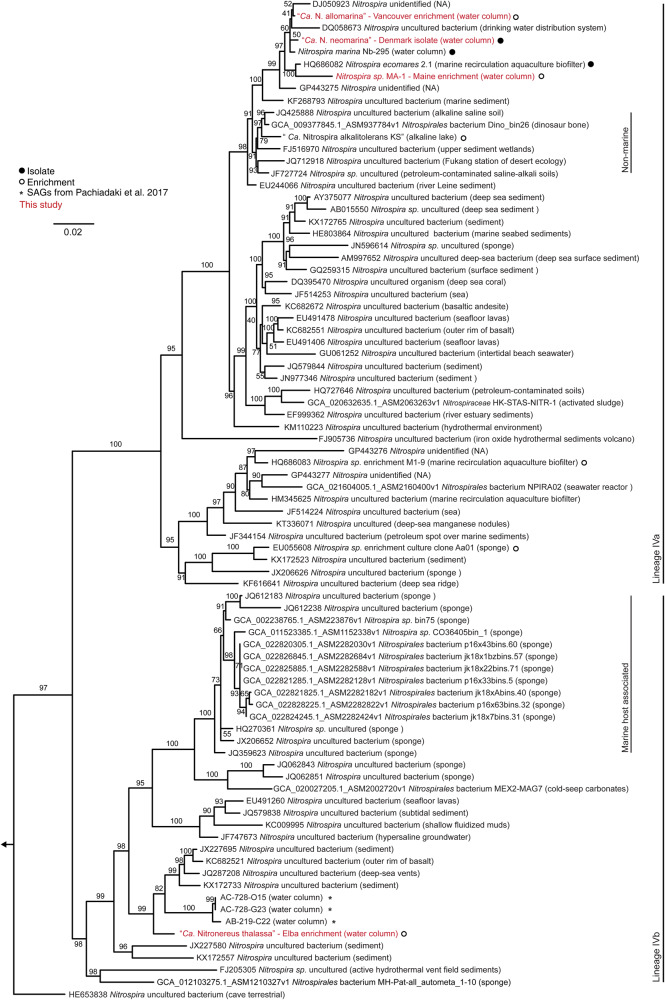
Phylogeny of lineage IV *Nitrospirales* based on 16S rRNA genes. Maximum likelihood tree based on the 16S rRNA gene sequences of *Nitrospirales* in lineage IV. The newly cultured *Nitrospirales* described in this study are highlighted in red. Isolated or enriched organisms are indicated by full and open circles, respectively, and SAGs from Pachiadaki et al. [47] are marked with an asterisk. Please refer to Materials and Methods for details of the sequence dataset used to calculate this tree. *Nitrospira moscoviensis* (lineage II) was used as outgroup. Numbers on the branches indicate ultra-fast bootstrap support (*n* = 1000). Sample sources are shown in parentheses. Lineages IVa and IVb are marked as described elsewhere [44]. The scale bar shows 0.02 estimated substitutions per nucleotide.

**Fig. 3:**
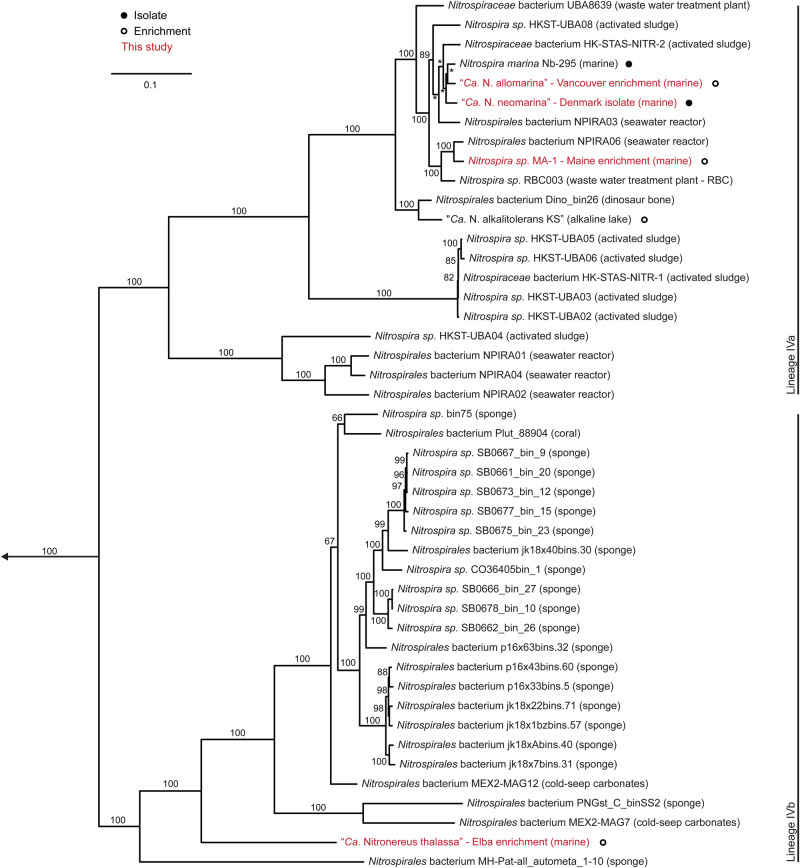
Phylogeny of lineage IV *Nitrospirales* based on 120 conserved proteins. Maximum likelihood tree based on a concatenated alignment of 120 conserved bacterial proteins from representative lineage IV *Nitrospirales* genomes and MAGs. The newly cultured marine *Nitrospirales* described in this study are highlighted in red. Isolated or enriched organisms are indicated by full and open circles, respectively. Please refer to Materials and Methods and Table S2 for details of the sequence dataset used to calculate this tree. The tree is excerpt from a more encompassing tree of the *Nitrospirales* (Fig. S3). The *Nitrospirales* outside of lineage IV are used as outgroup. Numbers on the branches indicate ultra-fast bootstrap support (*n* = 1000). Asterisks indicate a bootstrap value of 100. Sample sources are shown in parentheses. The scale bar shows 0.1 estimated substitutions per amino acid.

Within the genus *Nitrospira*, the phylogenetic lineage IV contains the marine *Nitrospira* members and is clearly distinguished from several other, mostly terrestrial and limnic *Nitrospira* lineages [5, 10, 14]. Furthermore, two distinct clades within lineage IV were previously described as sub-lineages IVa and IVb [44]. Until now, all cultured marine *Nitrospira*, including the type strain *N. marina* Nb-295 [9], belong to sub-lineage IVa (Fig. 2) [44]. A 16S rRNA-based phylogenetic analysis revealed that three of the four new marine NOB cultivated in this study (the Maine and Vancouver enrichments and the Denmark isolate) also cluster with lineage IVa (Fig. 2). The Vancouver and Denmark NOB are closely related to *N. marina* Nb-295 based on their 16S rRNA identities (Fig. S1). The nitrite oxidizer in the Maine enrichment is more closely related to *N. ecomares 2.1* (Fig. S1), an isolate from a marine recirculating aquaculture system [14]. In contrast, the NOB from the Elba enrichment is only distantly related (below 93% 16S rRNA identity) to all cultured *Nitrospira* (Fig. 2, Fig. S1). This NOB is the first cultured member of lineage IVb (Fig. 2), which encompasses sponge associated and free-living organisms [5]. The phylogenetic affiliation of the newly cultured marine NOB was also analyzed by using a dataset of conserved concatenated marker protein sequences (Fig. 3). This dataset comprised the sequences obtained in this study and a curated dataset of publicly available *Nitrospirales* genomes (see Materials and Methods and Table S2). In the concatenated marker protein phylogeny, lineage IV was recovered as a monophyletic clade that was distinct from the terrestrial *Nitrospira* lineages (Fig. S3). It also confirmed that the Vancouver, Maine, and Denmark *Nitrospirales* are closely affiliated with *N. marina* Nb-295 [9, 20], whereas the Elba *Nitrospirales* shares a common ancestor with sponge-derived MAGs (Fig. 3, Fig. S3).

Genome-wide average nucleotide identity (gANI) (Fig. S4), average amino acid identity (AAI) (Fig. S5), and genome taxonomy database toolkit (GTDB-tk) [30, 45] based analyses were performed on the same *Nitrospirales* dataset to classify the newly cultured marine NOB on the species and genus levels and higher taxonomic ranks. Based on the gANI analysis and a species level threshold of 96.5 % [36], the four newly cultured NOB examined in this study are separate species from each other and *N. marina* Nb-295 (Fig. S4, Supplementary Results and Discussion). For the Denmark isolate and the Vancouver enrichment *Nitrospira*, we therefore tentatively propose the names “*Candidatus* Nitrospira neomarina DK” and “*Candidatus* Nitrospira allomarina VA”, respectively. We refrain from proposing a new species name for the *Nitrospira* in the Maine enrichment, since no genome is available for *N. ecomares* 2.1 [14], which shares a very high 16S rRNA identity (99.8%) with the *Nitrospira* from the Maine enrichment (Fig. S1). Henceforth, we refer to this organism as *Nitrospira sp*. MA-1. The AAI and GTDB-tk (Fig. S5) analyses corroborated the phylogenetic placement (Fig. 3) of the Elba *Nitrospirales* with the sponge-associated MAGs and further suggested that it represents a novel genus of marine NOB for which we tentatively propose the name “*Candidatus* Nitronereus thalassa EB” (Supplementary Results and Discussion). Moreover, according to both AAI analysis and the current GTDB classification, *Nitrospira* lineages I and II may actually be members of a different family than the marine *Nitrospira* [46] (Fig. S5, Table S2). Hence, our analyses suggest that the taxonomy of *Nitrospira* may need a thorough reevaluation (Supplementary Results and Discussion), which would however be beyond the scope of this study focusing on the characterization of newly cultured marine NOB. To reflect the observed discrepancy between the molecular data and the current naming convention, we will from here on refer specifically to a part of lineage IVa (including the type strain *N. marina* Nb-295, the Denmark isolate, and the Vancouver and Maine enrichment NOB, see also Fig. S5) as genus *Nitrospira* and to the remaining organisms as members of the order *Nitrospirales*. Consistently, we also use the term *Nitrospirales* when referring collectively to all the mentioned organisms. Established names of species like *Nitrospira moscoviensis* (lineage II), however, are used here without change.

### Global abundance of marine *Nitrospirales*

The abundance and distribution of NOB across different marine habitats was assessed previously based on a mapping of marine metagenomic sequence reads to a selection of NOB genomes, MAGs, and SAGs [47]. Those results showed that marine *Nitrospinales* “Clade 1” and “Clade 2” [47] were the most abundant NOB in the majority of the samples. However, the few *Nitrospirales* SAGs included in that analysis were also abundant or even dominant in some of the marine metagenomes. Based on their 16S rRNA genes, those uncultured *Nitrospirales* SAGs are related to “*Ca*. Nitronereus thalassa EB” enriched in our present study (Fig. 2, Fig. S1). Here, we used the IMNGS tool [38] to further explore the environmental abundance of representatives of marine NOB based on 16S rRNA gene frequencies in publicly available amplicon datasets. We focused on the *Nitrospirales* and *Nitrospinales* and used as reference organisms *N. marina* Nb-295 for *Nitrospira* (lineage IVa), “*Ca*. Nitronereus thalassa EB” for *Nitrospirales* lineage IVb, and selected *Nitrospinales* members (Tables S3 and S4). We excluded the NOB genus *Nitrobacter*, which contains only a few marine species and was already known to occur at very low abundances in marine sequence datasets [47]. We also excluded the marine genus *Nitrococcus* that has versatile metabolic activities outside nitrification and whose abundance patterns were found to not correlate with in situ nitrite oxidation rates [48].

**Fig. 4:**
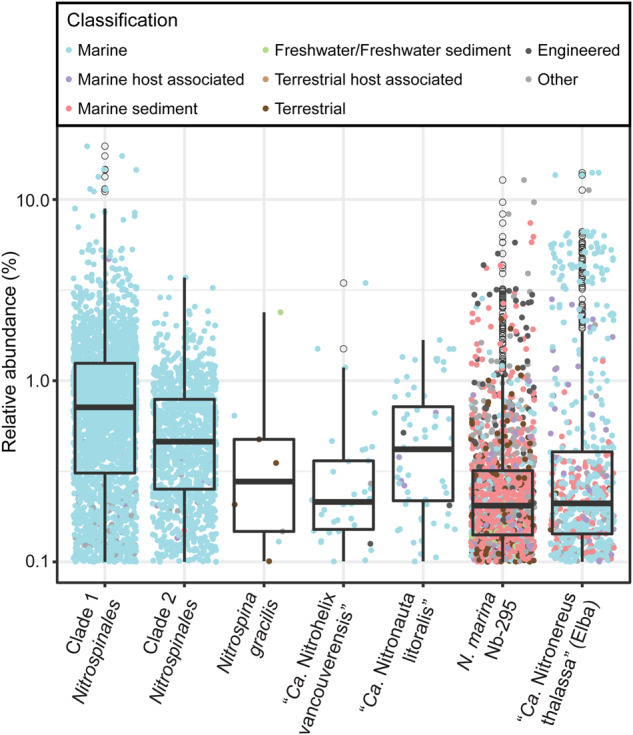
Environmental distribution of lineage IV *Nitrospirales* and *Nitrospinales*. Relative abundances of 16S rRNA genes grouped at 97% identity level from cultured and uncultured lineage IV *Nitrospirales* and *Nitrospinales* taken from publicly available amplicon sequencing datasets. The 16S rRNA gene-based abundance values were obtained from IMNGS. Each data point represents an environmental sample where the respective clade or strain, or closely related organisms, was detected with a minimal relative abundance of 0.1%. Colors represent different source environments.

Our analysis revealed distinct habitat distributions of the different marine NOB. Consistent with earlier findings [47, 49], the uncultured Clade 1 *Nitrospinales* represented the most frequently found NOB in the marine water column. It showed up to 20% relative abundance in the respective microbial 16S rRNA sequence datasets (Fig. 4). This clade, and also the highly abundant and uncultured *Nitrospinales* Clade 2, were almost exclusively detected in water column samples (Fig. 4). Contrasting Clades 1 and 2, organisms closely related to the cultured *Nitrospinales* members turned out to be the least frequently detected NOB in our analysis (Fig. 4). Among the groups containing cultured NOB, *Nitrospira* (lineage IVa containing *N. marina* Nb-295, the Denmark isolate and the Maine and Vancouver enrichment NOB) was most frequently encountered. However, this lineage was mainly distributed in marine sediments and occurred also frequently and to high relative abundance in various non-marine and engineered environments, such as soils and wastewater treatment plants (Fig. 4). Lineage IVa *Nitrospira* had previously been considered to occur virtually exclusively in marine habitats [2, 50] but were also recently found in saline-alkaline lakes [13]. However, their presence in freshwater-based engineered systems, such as a municipal wastewater treatment plant [51], was unexpected (Fig. 3 and 4). Currently, *Nitrospira* lineage IVa appears to be the only major marine NOB group that is also regularly found outside marine ecosystems. Thus, more insight into their ecophysiology will be needed to explain their wide environmental distribution and to obtain a more complete picture of the niche partitioning among nitrifying microorganisms in a broad range of habitats.

*Nitrospirales* lineage IVb (containing “Ca. Nitronereus thalassa EB”) was also abundant but, in contrast to *Nitrospira* lineage IVa, its members occurred mostly in the marine water column and in host-associated marine communities (Fig. 4). In sponges, lineage IVb *Nitrospirales* were the dominant known NOB, and they reach relative abundances of up to 14% in the water column (Fig. 4 and Table S4). These results are congruent with the literature [5, 6] and our observation that lineage IVb encompasses both free-living and sponge-associated *Nitrospirales* (Figs. 2, 3).

### Genomic potential of the newly cultured *Nitrospirales*

A pan-genome analysis of the four newly cultured *Nitrospirales* and of *N. marina* Nb-295 (Fig. S6) showed that the shared gene content of these five marine *Nitrospirales* members reflects their phylogenetic relationships. Although “*Ca*. Nitronereus thalassa EB” from lineage IVb has the smallest genome, it contains more unique genes (1431) than any of the four lineage IVa genomes. A large number of genes (>1000) is present in all lineage IVa *Nitrospira*, whereas these genes are absent from the genome of the ”*Ca*. Nitronereus thalassa”. The three phylogenetically most closely related *Nitrospira* (Vancouver, Denmark, and *N. marina* Nb-295) share a large core genome (Fig. S6).

### Nitrogen and carbon metabolism

**Fig. 5:**
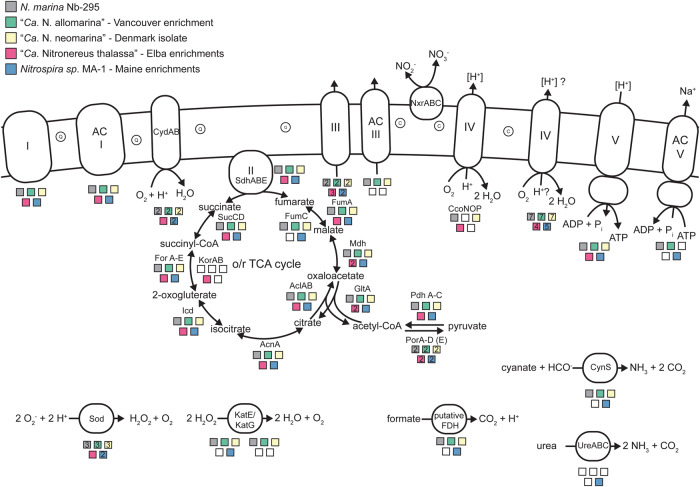
Selected key metabolic pathways in the newly cultured marine *Nitrospirales* and *N. marina*-Nb-295. Schematic illustration of the genetic inventory for the membrane-bound electron transport chain, the oxidative and reductive TCA cycles, and other selected pathways in the newly cultivated *Nitrospirales* and *N. marina* Nb-295. The colored squares indicate the presence of the genes in the respective genomes, with numbers indicating gene copy numbers if more than one copy is present. Please refer to Table S6 for a complete list of gene abbreviations and details of the gene annotations. Q quinone, C *c*-type cytochrome, AC alternative complex, CcoNOP cyt. *cbb*_*3*_ terminal oxidase, o/r TCA cycle oxidative and reductive tricarboxylic acid cycle. Roman numbers indicate the respiratory chain complexes.

As expected, the hallmark genes for chemolithoautotrophic nitrite oxidation and CO_2_ fixation are shared between all the newly cultured *Nitrospirales* and *N. marina* Nb-295. This core genetic repertoire includes the genes encoding the known subunits of nitrite oxidoreductase (*nxrABC*), the oxidative (oTCA) and the reductive (rTCA) tricarboxylic acid cycles, and the membrane-bound respiratory chain (Fig. 5, Table S5). However, “*Ca*. Nitronereus thalassa EB” harbors a unique genomic region with an extra copy each of two TCA cycle genes, citrate synthase (*gltA*) and malate dehydrogenase (*mdh*). A hallmark enzyme of the rTCA cycle in *Nitrospira* is a five-subunit form of 2-oxalogluterate:ferredoxin oxidoreductase (For), which is thought to work as a bidirectional enzyme and should thus function also in the oTCA cycle in *Nitrospirales* [52]. In addition to the five *forA-E* genes, the aforementioned genomic locus of “*Ca*. Nitronereus thalassa EB” encodes also an isoenzyme (Kor) that consists of only two subunits, KorAB (Fig. 5). A mutant study on the *for* and *kor* genes in *Hydrogenobacter thermophilus*, a hydrogen-oxidizing chemolithoautotroph, showed that the presence of the more oxygen-tolerant For improved growth yield under oxic conditions, whereas Kor was necessary for anaerobic growth [53]. Hence, the presence of both the *for* and the *kor* genes in “*Ca*. Nitronereus thalassa EB” might indicate more flexibility with regard to different oxygen conditions. As the *kor* genes were not found in any other lineage IV *Nitrospirales* or MAG that we analyzed (Fig. S7, Table S7), it is tempting to speculate that Kor could be a specific adaptation of “*Ca*. Nitronereus thalassa EB” to low-oxygen or even anoxic conditions. In basal *Nitrospirota* clades, *kor* genes were also found and predicted to have been present in the last common ancestor of the *Nitrospirota*, which likely inhabited anoxic environments [54].

*Nitrospirales* genomes commonly contain a presumably oxygen-tolerant, five-subunit form of pyruvate:ferredoxin oxidoreductase (Por) [52]. In addition, all four of the new *Nitrospirales* harbor a putative four-subunit form of Por, which has thus far only been found in lineage IVa *Nitrospira* but not in other *Nitrospirales* members [13, 20]. The expression levels of this Por increased strongly in microoxic growth conditions in *N. marina* Nb-295, suggesting that the four-subunit Por also represents an adaptation to low-oxygen environments [20].

Respiratory complex I seems to be conserved among the new marine *Nitrospirales* and *N. marina* Nb-295, including an alternative complex I [52]. This alternative form, which contains two M-subunits (instead of only one in the canonical complex I), is widespread in marine and terrestrial *Nitrospirales* genomes. It might use proton motive force for the reverse transport of electrons from quinol to the low-potential ferredoxins needed in the rTCA cycle [55].

More differences were observed with regard to the complexes III, IV, and V of the respiratory chain. Two versions of the canonical respiratory complex III occur in all the newly cultured marine NOB and were postulated to function in forward and reverse electron transport in *Nitrospirales* [52]. “*Ca*. Nitronereus thalassa EB” further encodes a third copy of the canonical complex III in the same genomic region as the additional TCA cycle genes *gltA*, *mdh*, and *korAB* (Fig. 5, Table S6). Moreover, the genome of *N. marina* Nb-295 harbors a putative alternative complex III, ACIII [20]. ACIII also occurs in “*Ca*. N. neomarina DK” and “*Ca*. N. allomarina VA” and in several lineage IVa MAGs but is absent in the *Nitrospira sp*. MA-1 and “*Ca*. Nitronereus thalassa EB” (Fig. 5, Fig. S7). This type of alternative complex may be involved in linear or bifurcated electron transfer from quinol (or another suitable electron donor) to either one- or two-electron acceptors possibly coupled to energy conservation [56]. As the *Nitrospirales* are equipped with different copy numbers and types of complex III, they likely differ in the regulation and biochemistry of electron transport as part of their core metabolism.

In *Nitrospirales*, a putative novel cytochrome (cyt.) *bd*-like terminal oxidase is thought to act as the main cyt. *c*-oxidizing terminal oxidase (complex IV), since canonical heme-copper terminal oxidases are absent from most *Nitrospirales* genomes [52]. The marine *Nitrospirales* genomes analyzed in this study possess multiple copies of these putative cyt. *bd*-like terminal oxidases (Table S6), including homologs of the terminal oxidase proposed for *Nitrospira defluvii* (Nide0901) [52], and of the cyt. *bd*-like terminal oxidase proposed for NOB from the *Nitrospinales* [25]. Furthermore, all newly cultured *Nitrospirales* encode at least one canonical cyt. *bd* quinol oxidase (genes *cydAB*) (Fig. 5, Table S6) that could accept electrons stemming from electron donors with a lower reduction potential than NO_2_^-^, such as formate, hydrogen, or glycogen [17–19, 52]. Several of the lineage IVa *Nitrospira* and IVb *Nitrospirales* are unique in their terminal oxidase collection, as they also have a canonical heme-copper cyt. *cbb*_3_ terminal oxidase (Fig. 5, Fig. S7). In *N. marina* Nb-295, this *cbb*_3_ terminal oxidase was upregulated under low O_2_ conditions, indicating that it may be advantageous under oxygen limitation. This function would be consistent with the known high affinity for O_2_ of *cbb*_3_ terminal oxidases in other organisms [57]. However, the lack of a *cbb*_3_ terminal oxidase in “*Ca*. N. allomarina VA”and *Nitrospira sp*. MA-1 (Fig. 5, Fig. S7) suggests that even closely related marine *Nitrospira* may differ in their adaptation to O_2_-limited conditions. Lastly, we observed differences in the repertoire of ATPases (complex V). All four *Nitrospirales* possess a canonical F_1_F_O_-ATPase. However, only “*Ca*. N. allomarina VA” and *Nitrospira sp*. MA-1 also encode a putative Na^+^-translocating N-type ATPase (Fig. 5), which is present in *N. marina* Nb-295, “*Candidatus* Nitrospira alkalitolerans”, and several other lineage IVa *Nitrospira* (Fig. S7). It might contribute to salt resistance and the generation of sodium motive force [13, 20]. Multiple subunits of the N-ATPase were also significantly upregulated at low O_2_ in *N. marina* Nb-295 [20].

### Organic nitrogen utilization and other alternative metabolisms

*Nitrospirales* have been shown to utilize a broad range of alternative substrates for energy generation and nitrogen assimilation [17, 19–23]. *N. marina* Nb-295 and the newly cultured *Nitrospirales*, except for “*Ca*. Nitronereus thalassa EB”, possess the cyanase gene (*cynS*) that facilitates the conversion of cyanate to ammonia and CO_2_ [21]. This feature is common across the *Nitrospira* and sponge-associated lineage IVb *Nitrospirales* (Fig. S7). *Nitrospira sp*. MA-1 further harbors a gene cluster encoding a urease (*ureABC*) (Fig. 5) and a urea transporter, which are also present in a closely related *Nitrospira* lineage IVa MAG from a wastewater treatment plant [51]. The urea transporter of *Nitrospira sp*. MA-1 differs completely from that encoded by *N. moscoviensis* (lineage II). *Nitrospira sp*. MA-1 harbors a one-subunit urea transporter that is homologs to mammalian urea transporters [58], whereas *N. moscoviensis* has an ABC-type transporter [19]. While the ability to use urea occurs only sporadically in lineage IVa members and is absent in “Ca. Nitronereus thalassa EB”, it is widely distributed among the sponge-associated *Nitrospirales* (Fig. S7). Both the cyanase and urease may allow the *Nitrospirales* to indirectly use cyanate and urea as energy sources *via* “reciprocal feeding” with ammonia-oxidizing microorganisms lacking these enzymes, as previously demonstrated for *N. moscoviensis* [19, 21]. Moreover, these enzymes likely enable *Nitrospirales* to utilize cyanate and urea for nitrogen assimilation, similar to uncultured marine *Nitrospinales* [59]. The genomic potential for cyanate and urea utilization seems to be widespread but not ubiquitous among marine *Nitrospirales* (Fig. 5, Fig. S7) [47]. It may be an important and niche-defining adaptation of these NOB to ammonia- or nitrite-deplete conditions in oligotrophic environments [59]. Marine comammox *Nitrospirales* have not been found to date. This is consistent with a recent, genome-based analysis of metabolic expansion and progression in the *Nitrospirota*, which indicated that the ability to perform ammonia oxidation was likely acquired *via* lateral gene transfer by the terrestrial comammox organisms after their divergence from the marine *Nitrospirales* [54].

The utilization of formate as an alternative energy and carbon source has been demonstrated for *N. moscoviensis* [19], *N. marina* Nb-295, and other NOB [60–62]. The three new marine lineage IVa *Nitrospira* possess a putative formate dehydrogenase (FDH; Fig. 5), which is widespread among environmental *Nitrospira* but seems to be absent in lineage IVb including “*Ca*. Nitronereus thalassa EB” (Fig. S7). The expression of FDH was upregulated at low O_2_ and it was proposed to catalyze formate oxidation in *N. marina* Nb-295 [20]. Upregulation of FDH at hypoxia may indicate that marine *Nitrospira* can use formate anaerobically with nitrate as the terminal electron acceptor, a metabolism already demonstrated for the non-marine *N. moscoviensis* [19]. In this case, nitrate reduction is most likely catalyzed by nitrite oxidoreductase running in reverse [19, 63]. Lastly, the four newly cultured *Nitrospirales* encode a group 3b NiFe hydrogenase [20, 22, 24, 25] and a putative sulfite cyt. *c* oxidoreductase (*sorAB*) [20, 22, 24, 25], which are commonly observed among the *Nitrospirales* and *Nitrospinales* although no phenotype has been linked yet to the presence of these enzymes in NOB.

### Stress response

Microorganisms living in oxic environments must cope with reactive oxygen species (ROS), such as superoxide and hydrogen peroxide. However, numerous *Nitrospirales* members and other NOB, such as *Nitrospinales*, lack superoxide dismutase (SOD), catalase, or even both of these key ROS-detoxifying enzymes [22–25, 52]. The new *Nitrospirales* members possess a comparatively large genomic repertoire for ROS detoxification. Their genomes contain one to three superoxide dismutase genes (*sod*) (Fig. 5, Table S6). A canonical heme catalase gene (*katE)* is present in the Maine, Vancouver, and Denmark *Nitrospira*, and an additional heme catalase peroxidase gene (*katG*) occurs in the “*Ca*. N. allomarina VA” and “*Ca*. N. neomarina DK” (Fig. 5, Table S6). Only “*Ca*. Nitronereus thalassa EB” lacks catalase and might thus be more susceptible to oxidative stress. However, all the newly cultured *Nitrospirales* contain also other enzymes, such as the canonical cyt. *bd* quinol oxidase (see above), glutaredoxins, and thioredoxins, which can contribute to H_2_O_2_ detoxification and occur in other *Nitrospirales* and in *Nitrospinales* [25, 52, 64]. The high salinity of seawater is another stress factor relevant to marine NOB. The production and/or acquisition of osmocompatible solutes, and the intracellular accumulation of ions, are the two main strategies to counteract osmotic stress. Betaine and ectoine are two of the major osmolytes produced by microorganisms [65]. The new marine *Nitrospirales* have the genomic potential to import glycine betaine using a glycine/betaine/choline ABC transporter OpuABD and a predicted high-affinity glycine betaine transporter homologous to OpuD of *Bacillus subtilis* [66, 67] and other bacteria. In addition, they should be able to synthesize betaine from glycine with a fused glycine/sarcosine N-methyltransferase and dimethylglycine N-methyltransferase (*bsmAB*) (Table S6). Betaine synthesis and import are also encoded by the terrestrial, alkalitolerant strain “*Ca*. N. alkalitolerans” but do not seem to be widespread in *Nitrospirales* outside lineage IV [13, 67]. Trehalose also seems to be employed as an osmolyte in marine *Nitrospirales* including the four newly cultured representatives (Table S6) [20, 67]. No genes for ectoine synthesis or import have been identified in any of the new marine *Nitrospirales* and *N. marina* Nb-295, contrasting the putatively widespread utilization of ectoine in the *Nitrospinales* [25, 49]. In addition to osmolytes, the new marine *Nitrospirales*, as well as *N. marina* Nb-295 and “*Ca*. N. alkalitolerans” [13, 20] appear to utilize several Na^+^ and H^+^ transporters to maintain intracellular ion homeostasis. Finally, all the newly cultured marine *Nitrospirales* encode genes for the squalene-hopene cyclase (*shc*), a key gene in hopanoid synthesis, and an adenosyl-hopene transferase (*hpnH*). Hopanoids are microbial lipids that can regulate membrane fluidity, similar to the role of cholesterol in eukaryotic membranes [68]. Suggested functions for hopanoids include temperature, pH, and osmotic stress regulation [69, 70]. The ability to synthesize different types of hopanoids seems to be widely distributed in marine and terrestrial NOB [25, 71–73]. The production of certain types of hopanoids might be linked to the availability of vitamin B_12_, which is a cofactor of the two hopanoid methylases HpnR and HpnP [72, 73]. *N. marina* Nb-295 and the Denmark and the Vancouver *Nitrospira* possess the hopanoid C-3 methylase gene *hpnR*. This is peculiar, because marine *Nitrospirales* are vitamin B_12_ auxotrophs based on the genomes analyzed here and on experimental evidence from *N. marina* Nb-295 [20].

## Conclusions

Microbial ecology has been moving beyond describing the diversity of complex microbial communities and toward deciphering the functions of the plethora of uncharacterized microorganisms detected by molecular tools. In this context, the cultivation of prevalent but generally intractable microorganisms is gaining importance. The *Nitrospirales* are ubiquitous and play important roles for nutrient cycling in most ecosystems, but they are infamously difficult to cultivate. In this study, we greatly increased the number of available cultured marine *Nitrospirales* members. Through the application of a recently developed isolation method, we were also able to rapidly purify a new *Nitrospira* strain. Furthermore, the enriched “*Ca*. Nitronereus thalassa EB” is the first cultured representative of the environmentally abundant (Fig. 4) lineage IVb *Nitrospirales*. Genome comparisons between the newly cultured marine NOB have provided insight into the core and flexible gene repertoires of the marine *Nitrospirales*, which can be used as a foundation for future experiments to further characterize these organisms. Three of the *Nitrospira* are closely related to *N. marina* Nb-295, but numerous genomic differences have been detected (Fig. 5, Fig. S7), which include prominent features such as the lack of a cyt. *cbb*_3_ terminal oxidase in the “*Ca*. N. allomarina VA” (Fig. 5). These NOB could thus serve as ‘natural knock-outs’ for comparative physiological studies, as there are currently no genetic tools available for manipulating *Nitrospirales*.

### Taxonomic consideration of “*Candidatus* Nitronereus thalassa” gen. nov. sp. nov

Ni.tro.ne’reus L. n. nitrum: nitrate, Gr. masc. n. nereus: a sea god from Greek mythology; N.L. masc. n. *Nitronereus* nitrate-forming sea god. tha’las.sa Gr. fem. n. *thalassa*: sea.

A nitrate-forming bacterium obtained from the Mediterranean Sea. Phylogenetically affiliated with the order *Nitrospirales*, phylum *Nitrospirota*. The genome consists of two scaffolds of in total 4,014,679 bp. The DNA G + C content is 48.5 mol%. “*Ca*. Nitronereus thalassa EB” was cultivated from coastal surface water from Elba, Italy. Marine aerobic chemolithoautotroph that oxidizes nitrite to nitrate. “*Ca*. Nitronereus thalassa EB” was routinely cultured with 0.25 mM nitrite at 28 °C in liquid marine mineral medium. Could not be grown on solid medium. Auxotrophic for vitamin B_12_ according to genome analysis.

### Taxonomic consideration of “*Candidatus* Nitrospira neomarina” sp. nov

Neo.ma.ri’na Gr. masc. adj. neos: new; N.L. fem. adj. ma.ri’na: of the sea; N.L. fem. adj. *neo.ma.ri’na*: a species related to but distinct from the existing species *Nitrospira marina*.

Phylogenetically affiliated with the genus *Nitrospira*, phylum *Nitrospirota*. The genome consists of one circular chromosome of 4,796,652 bp. The DNA G + C content is 50 mol%. Strain “*Ca*. Nitrospira neomarina DK” was cultivated from coastal surface water from Slettestrand, Denmark. Marine aerobic chemolithoautotroph that oxidizes nitrite to nitrate. The strain was routinely cultured with 1 mM nitrite at 28 °C in liquid sea salt based marine mineral medium with vitamin supplements. Could not be grown on solid medium. Auxotrophic for vitamin B_12_ according to genome analysis.

### Taxonomic consideration of “*Candidatus* Nitrospira allomarina” sp. nov

Allo.ma.ri’na Gr. masc. adj. allos: other; N.L. fem. adj. ma.ri’na: of the sea; N.L. fem. adj. *allo.ma.ri’na*: a species related to but distinct from the existing species *Nitrospira marina*.

Phylogenetically affiliated with the genus *Nitrospira*, phylum *Nitrospirota*. The genome consists of one circular chromosome of 4,553,057 bp. The DNA G + C content is 50 mol%. “*Ca*. Nitrospira allomarina VA” was cultivated from coastal surface water from Vancouver, Canada. Marine aerobic chemolithoautotroph that oxidizes nitrite to nitrate. “*Ca*. Nitrospira allomarina VA” was routinely cultured with 1 mM nitrite at 28 °C in liquid sea salt based marine mineral medium with vitamin supplements. Could not be grown on solid medium. Auxotrophic for vitamin B_12_ according to genome analysis.

### Supplementary information

Supplementary Information

Table S1

Table S2

Table S3

Table S4

Table S5

Table S6

Table S7

## Data Availability

The assembled *Nitrospirales* genomes and raw sequencing reads are available at NCBI BioProject PRJNA922051.
