# Supplementary material for: Cultivation and genomic characterization of novel and ubiquitous marine nitrite-oxidizing bacteria from the Nitrospirales
Source: ISME J. 2023 Sep 25;17(11):2123–33. doi: 10.1038/s41396-023-01518-6 (PMC10579370; doi:10.1038/s41396-023-01518-6)
Supplement: Supplementary file 1 — Supplementary Information [file 41396_2023_1518_MOESM1_ESM.pdf]

Cultivation and genomic characterization of novel and ubiquitous marine nitrite-oxidizing bacteria from the *Nitrospirales*

Anna J. Mueller, Anne Daebeler, Craig W. Herbold, Rasmus H. Kirkegaard, Holger Daims

Supplementary Materials and Methods

DNA extraction, sequencing, assembly, binning and annotation.

Biomass for DNA extraction was harvested from 20 to 300 ml aliquots of the cultures by centrifugation at 4500×g for 20 min at 4°C (swing-bucket rotors) and stored at -20°C until further processing. DNA for short-read sequencing (Illumina) was isolated from the collected biomass with the Qiagen Power Soil kit (Denmark isolate) and the Qiagen Power Soil Pro Kit (Vancouver, Maine, Elba enrichments) according to the manufacturer's protocols. DNA for long-read sequencing (Nanopore) was also isolated from all four cultures with the Qiagen Power Soil Pro Kit. Short-read sequencing for all four cultures (NovaSeq 6000, Illumina) and long-read sequencing (SQK-NBD112.24 barcoding kit, R10.4 flowcell, on a MinION, Oxford Nanopore Technologies) for the Elba, Maine, and Vancouver cultures were performed at the Joint Microbiome Facility Vienna (https://jmf.csb.univie.ac.at/) including quality control and library preparation. For the Denmark isolate, long-read sequencing (MinION, Oxford Nanopore Technologies), DNA quality control and library preparation were performed by the Vienna Biocenter Sequencing Core Facility NGS Unit (www.viennabiocenter.org/facilities). To obtain the best possible MAGs or genomes of each *Nitrospirales*, different assembly and binning methods were applied. The quality of the genomes was assessed by contamination and completeness as determined by CheckM (v1.2.0)[1]. The Illumina reads were converted from BAM to FASTQ files with Samtools (v1.15.4) [2]. Nanopore basecalling was performed with

Guppy (v. 6.1.1) with super accuracy mode (Elba, Maine Vancouver cultures) and demux (Denmark isolate). A closed circular genome of the Vancouver *Nitrospira* was assembled by flye (v2.9-b1768) with nano-hq [3] from the long Nanopore reads followed by three rounds of polishing with minimap2 (v2.17) [4] and racon (v1.4.3) [5] and then by two rounds of polishing with medaka (v1.6.0) (github.com/nanoporetech/medaka). Finally, the genome was further polished with the Illumina data using minimap2 (v2.17) and racon (v1.4.3). For the remaining *Nitrospirales* genomes, Illumina and Nanopore (flag –nanopore) hybrid assemblies were performed with Spades (v3.15.3 and v3.15.4). Assembly was performed for the Denmark isolate with the --isolate option, for the Maine enrichment with the –meta option, and for the Elba enrichment without any options except the default settings. A closed circular genome of the Denmark isolate was obtained after the Spades assembly. The remaining two assemblies were subjected to binning by Metabat (v 2.15), which used coverage information obtained from the Illumina reads mapped with bbmap (v 38.92), and finally the bins were subjected to dRep (v1.4.3). Based on the CheckM assessment, a MAG binned by Metabat 2 was selected for the Elba enrichment and a MAG binned with Metabat 1 (parameters 95_90) for the Maine enrichment. The genomes and MAGs were annotated by using the MicroScope platform (v3.16.0) [6] as described previously [7, 8]. Our lineage IV *Nitrospirales* genomic dataset was screened for the presence of specific proteins of interest (Table S7) with blastP (v 2.13.0) and the setting: -max_target_seqs 1. Thresholds of 50% for the alignment length (query length/alignment length) and of 35% for the amino acid sequence identity were applied. The data was analyzed and visualized with the R package tidyverse (v1.3.2) and edited in Adobe Illustrator.

FISH and CARD-FISH. Aliquots of the *Nitrospirales*-containing enrichment cultures and the Denmark isolate were fixed for 30 min in 3% paraformaldehyde at room temperature [9], washed twice in phosphate-buffered saline (PBS), and re-suspended in 1:1 PBS and ethanol (v/v). The *Nitrospirales* cells on the filters were visualized by rRNA-targeted FISH [9] (Elba, Maine, Vancouver enrichments) or CARD-FISH [10] (Denmark isolate) with the *Nitrospira*-specific, 16S rRNA-targeted oligonucleotide probe Ntspa662 [11], which was either double-labelled with the fluorophore Cy3 for FISH [12] or labelled with horseradish peroxidase (HRP) for CARD-FISH. Signal amplification by CARD-FISH was required to visualize the cells of the Denmark isolate, because FISH with double-labelled probes yielded only dim fluorescence signals with this culture at the time of sampling for the FISH/CARD-FISH experiments. A sequence alignment to the probe Ntspa662 confirmed that none of the 16S rRNA sequences of the four cultured *Nitrospirales* in this study had any nucleotide mismatches at the probe binding site. For FISH, a nonsense probe double-labelled with Cy5 and a probe mix binding to most bacterial cells (EUB338 mix I-III) [13] double-labelled with FLUOS were used in addition to the *Nitrospira*-specific probe Ntspa662. FISH was conducted according to standard protocols. Briefly, the fixed cells were dried on a microscope side and sequentially dehydrated in 50%, 70% and 100% (v/v) ethanol. The probes were hybridized in hybridization buffer containing 35% (v/v) formamide overnight at 46 °C, and washed for 10 min in washing buffer [9]. Finally, the cells were washed in ice-cold MilliQ water. CARD-FISH was conducted according to a standard protocol [10]. Specifically, the cells were immobilized on a microscope slide and dehydrated in 100% (v/v) ethanol, followed by the inactivation of endogenous peroxidases with 0.01 M HCl for 10 min at room temperature. The cells were hybridized in hybridization buffer containing 35% (v/v) formamide and the HRP-labelled probe for 3 h at 46 °C, and washed for 5 min in washing buffer

[14]. HRP equilibration was conducted for 15 min in 1×PBS at room temperature, and signal amplification was performed with Oregon Green 488-labelled tyramides (Thermo Fisher/ Life Science #O6147, 5mg) for 30 min at 46 °C and concluded with a final washing step for 15 min in 1×PBS at 48 °C. After FISH or CARD-FISH, the cells were additionally stained with DAPI (10 µg/ml) for 5 min and visualized using a Leica TCS SP8X confocal laser scanning microscope. The ImageJ Fiji [15, 16] application was used to overlay the images and to adjust their brightness and contrast.

Supplementary Results and Discussion

Nitrospirales Taxonomy

The genome wide average nucleotide identity (gANI) and average amino acid identity (AAI) analyses (Fig. S4) that were performed on the *Nitrospirales* genomes revealed that the Denmark, Vancouver, and Maine NOB belong to the genus *Nitrospira* (Fig. S5) but are separate species from each other and *N. marina* Nb-295 (main text, Fig. S4) [17]. With a gANI of only 72% to the most similar MAG (*Nitrospirales* bacterium MEX2-MAG12), the Elba NOB represents a new *Nitrospira*-like species not closely related to any other cultured organism. Since gANI values below 75% are considered unsuitable for taxonomic inferences [18], we considered average amino acid identities (AAI) and the genome taxonomy database (GTDB) [19] with the companion classification tool (GTDB-tk) [20] to elucidate the affiliation of the newly cultured NOB at the genus level and beyond. According to a genus level threshold of 60% AAI [18], lineage IV would encompass three separate genera based on the current dataset (Fig. S5). Two of these potential genera would correspond to lineage IVa, and one genus to IVb, which consistently are separate monophyletic groups (main text, Fig. 2, Fig. 3). Furthermore, while the

AAI cut-off allowed the Elba *Nitrospirales* and uncultured sponge-related MAGs in lineage IVb to be clustered into one genus (Fig. S5), GTDB-tk classified the Elba *Nitrospirales* as a separate genus from the sponge-related MAGs (Fig. S5, Table S2). Since the Elba *Nitrospirales* is the first cultured representative of a new genus, we propose to name it "*Candidatus* Nitronereus thalassa EB".

Furthermore, our gANI (Fig. S4), AAI (Fig. S5) and GTDB-tk (Table S2) analyses suggest that the currently recognized genus *Nitrospira* could be split into different genera or even families. Considering that the *Nitrospira* type strain, *N. marina* Nb-295 [17], is a member of the marine *Nitrospira* lineage IVa, only a subset of this lineage would likely retain the established genus name. Clearly, the taxonomy of *Nitrospira* (including the non-marine members) will need a thorough reevaluation.

References

1. Parks DH, Imelfort M, Skennerton CT, Hugenholtz P, Tyson GW. CheckM: Assessing the quality of microbial genomes recovered from isolates, single cells, and metagenomes. *Genome Res* 2015; **25**: 1043–1055.
2. Li H, Handsaker B, Wysoker A, Fennell T, Ruan J, Homer N, et al. the sequence alignment/map format and SAMtools. *Bioinformatics* 2009; **25**: 2078–2079.
3. Kovárová-Kovar K, Egli T. Growth kinetics of suspended microbial cells: from single-substrate-controlled growth to mixed-substrate kinetics. *Microbiol Mol Biol Rev* 1998; **62**: 646–66.
4. Li H. Minimap2: Pairwise alignment for nucleotide sequences. *Bioinformatics* 2018; **34**: 3094–3100.

5. Vaser R, Sović I, Nagarajan N, Šikić M. Fast and accurate de novo genome assembly from long uncorrected reads. *Genome Res* 2017; **27**: 737–746.

6. Vallenet D, Calteau A, Dubois M, Amours P, Bazin A, Beuvin M, et al. MicroScope: an integrated platform for the annotation and exploration of microbial gene functions through genomic, pangenomic and metabolic comparative analysis. *Nucleic Acids Res* 2020; **48**: D579–D589.

7. Lücker S, Wagner M, Maixner F, Pelletier E, Koch H, Vacherie B, et al. A *Nitrospira* metagenome illuminates the physiology and evolution of globally important nitrite-oxidizing bacteria. *Proc Natl Acad Sci U S A* 2010; **107**: 13479–13484.

8. Mueller AJ, Jung MY, Strachan CR, Herbold CW, Kirkegaard RH, Wagner M, et al. Genomic and kinetic analysis of novel Nitrospinae enriched by cell sorting. *ISME J* 2021; **15**: 732–745.

9. Holger Daims, Kilian Stoecker MW. Fluorescence in situ hybridization for the detection of prokaryotes. In: Osborn A. M. SCJ (ed). *Advanced Methods in Molecular Microbial Ecology*. 2005. Bios-Garland, Abingdon, U.K, pp 213–239.

10. Pernthaler A, Pernthaler J, Amann R. Fluorescence in situ hybridization and catalyzed reporter deposition for the identification of marine bacteria. *Appl Environ Microbiol* 2002; **68**: 3094–3101.

11. Daims H, Nielsen JL, Nielsen PH, Schleifer KH, Wagner M. In situ characterization of *Nitrospira*-like nitrite-oxidizing bacteria active in wastewater treatment plants. *Appl Environ Microbiol* 2001; **67**: 5273–5284.

12. Stoecker K, Dorninger C, Daims H, Wagner M. Double labeling of oligonucleotide probes for fluorescence *in situ* hybridization (DOPE-FISH) improves signal intensity and

increases rRNA accessibility. *Appl Environ Microbiol* 2010; **76**: 922–926.

13. Daims H, Brühl A, Amann R, Schleifer K-H, Wagner M. The domain-specific probe EUB338 is insufficient for the detection of all bacteria: development and evaluation of a more comprehensive probe set. *Syst Appl Microbiol* 1999; **22**: 434–444.
14. Manz W, Amann R, Ludwig W, Wagner M, Schleifer K-H. Phylogenetic oligodeoxynucleotide probes for the major subclasses of Proteobacteria: problems and solutions. *Syst Appl Microbiol* 1992; **15**: 593–600.
15. Schneider CA, Rasband WS, Eliceiri KW. NIH Image to ImageJ: 25 years of image analysis. *Nat Methods* 2012; **9**: 671–675.
16. Schindelin J, Arganda-Carreras I, Frise E, Kaynig V, Longair M, Pietzsch T, et al. Fiji: An open-source platform for biological-image analysis. *Nat Methods* 2012; **9**: 676–682.
17. Watson SW, Bock E, Valois FW, Waterbury JB, Schlosser U. *Nitrospira marina* gen. nov. sp. nov.: a chemolithotrophic nitrite-oxidizing bacterium. *Arch Microbiol* 1986; **144**: 1–7.
18. Rodriguez-R LM, Konstantinidis KT. Bypassing cultivation to identify bacterial species. *Microbe* 2014; **9**: 111–118.
19. Parks DH, Chuvochina M, Chaumeil PA, Rinke C, Mussig AJ, Hugenholtz P. A complete domain-to-species taxonomy for Bacteria and Archaea. *Nat Biotechnol* 2020; **38**: 1079–1086.
20. Parks DH, Chuvochina M, Waite DW, Rinke C, Skarshewski A, Chaumeil PA, et al. A standardized bacterial taxonomy based on genome phylogeny substantially revises the tree of life. *Nat Biotechnol* 2018; **36**: 996–1004.
21. Maixner F. The ecophysiology of nitrite-oxidizing bacteria in the genus *Nitrospira*: novel aspects and unique features. 2009. Universität Wien.

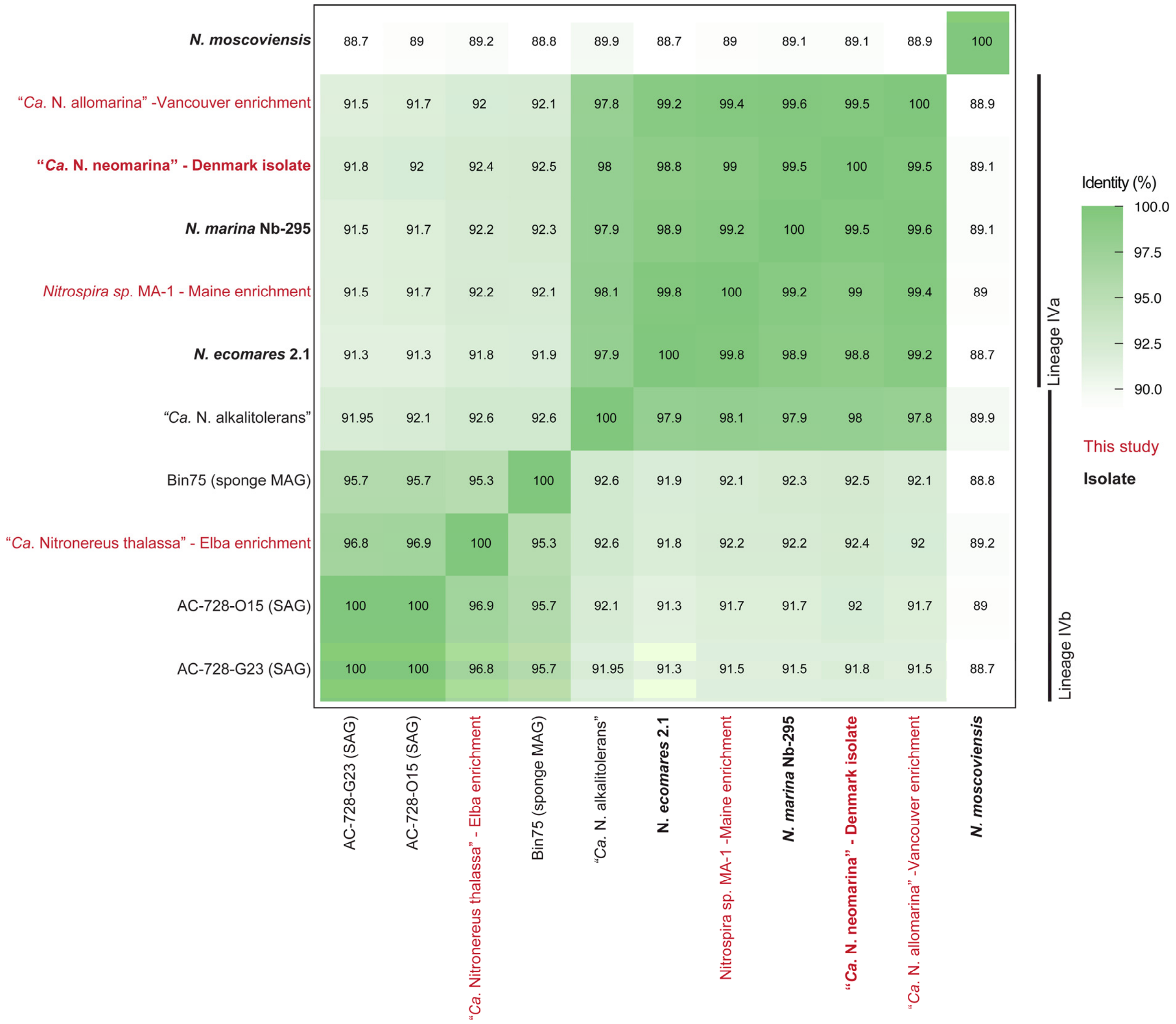

Figure S1. 16S rRNA gene identities of lineage IV *Nitrospirales*. Pairwise 16S rRNA gene (length >1500 nt) identities were determined by BLASTn. The organisms are ordered by their placement in the phylogenetic tree in main text Fig. 2. *Nitrospira moscoviensis* from lineage II is included for reference. Numbers in the squares are the 16S rRNA gene identities in percent.

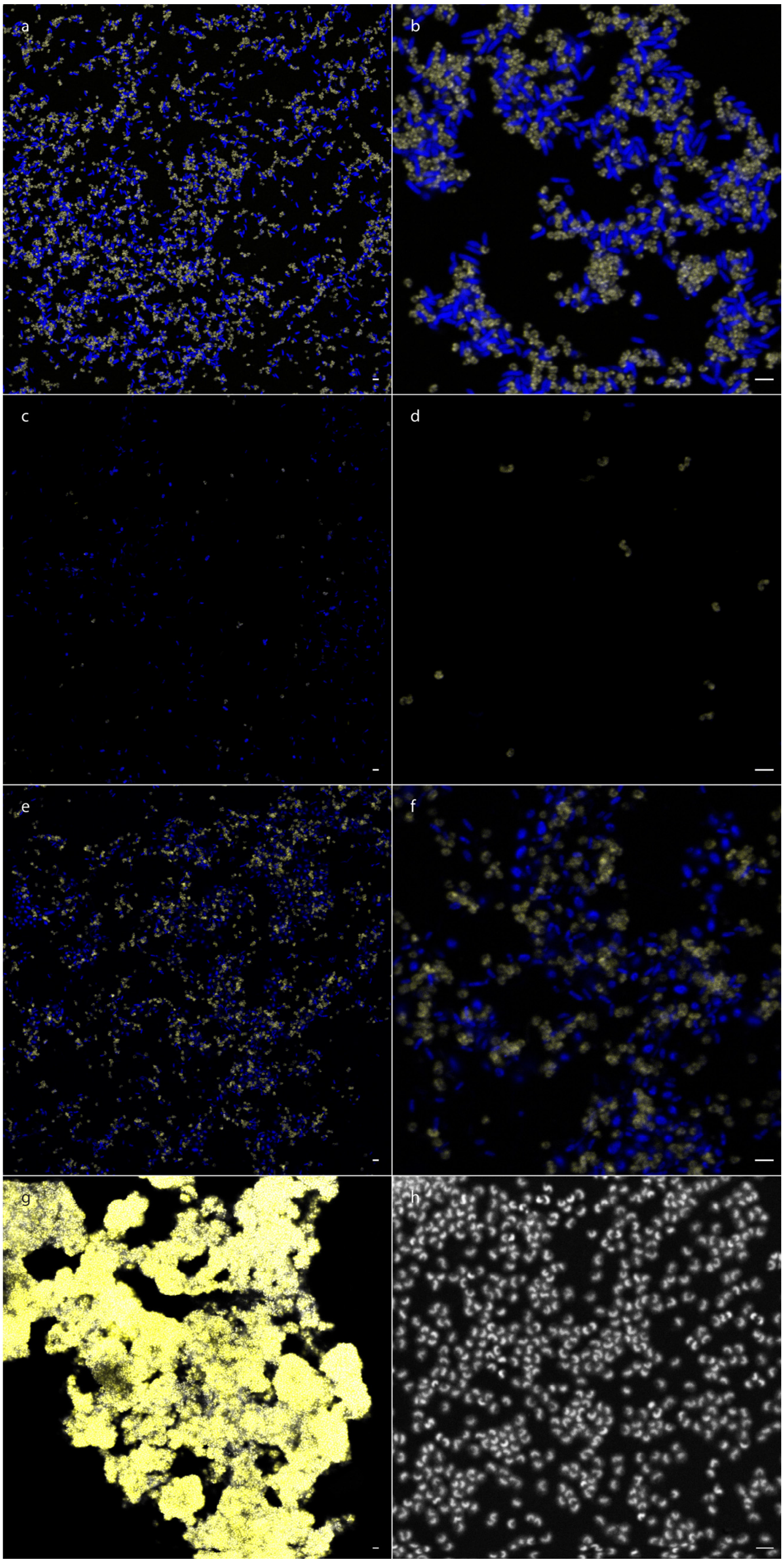
a
b
c
d
e
f
g
h

Figure S2. Fluorescence *in situ* hybridization of the new *Nitrospirales* cultures. Images show the *Nitrospirales*-containing enrichments from (a, b) Elba, (c, d) Maine, and (e, f) Vancouver labelled by 16S rRNA-targeted FISH with double-labelled probes Ntspa662 specific for *Nitrospirales* (Cy3; false-colored as yellow) and the EUB338 probe mix specific for *Bacteria* (FLUOS, false-colored as blue). Since *Nitrospirales* cells were labelled by all applied probes they appear in yellow to grey, whereas other bacteria appear in blue. Panels b, d, and f show the respective cultures at a higher magnification. Panel g shows the Denmark isolate labelled by CARD-FISH with probe Ntspa662 (Oregon Green 488, false-colored as yellow) and DAPI (false-colored as grey). Panel h shows the Denmark isolate labelled by DAPI at a higher magnification. In all images, brightness and contrast were adjusted for the whole images for illustration purposes. The scale bars indicate 2 µm.

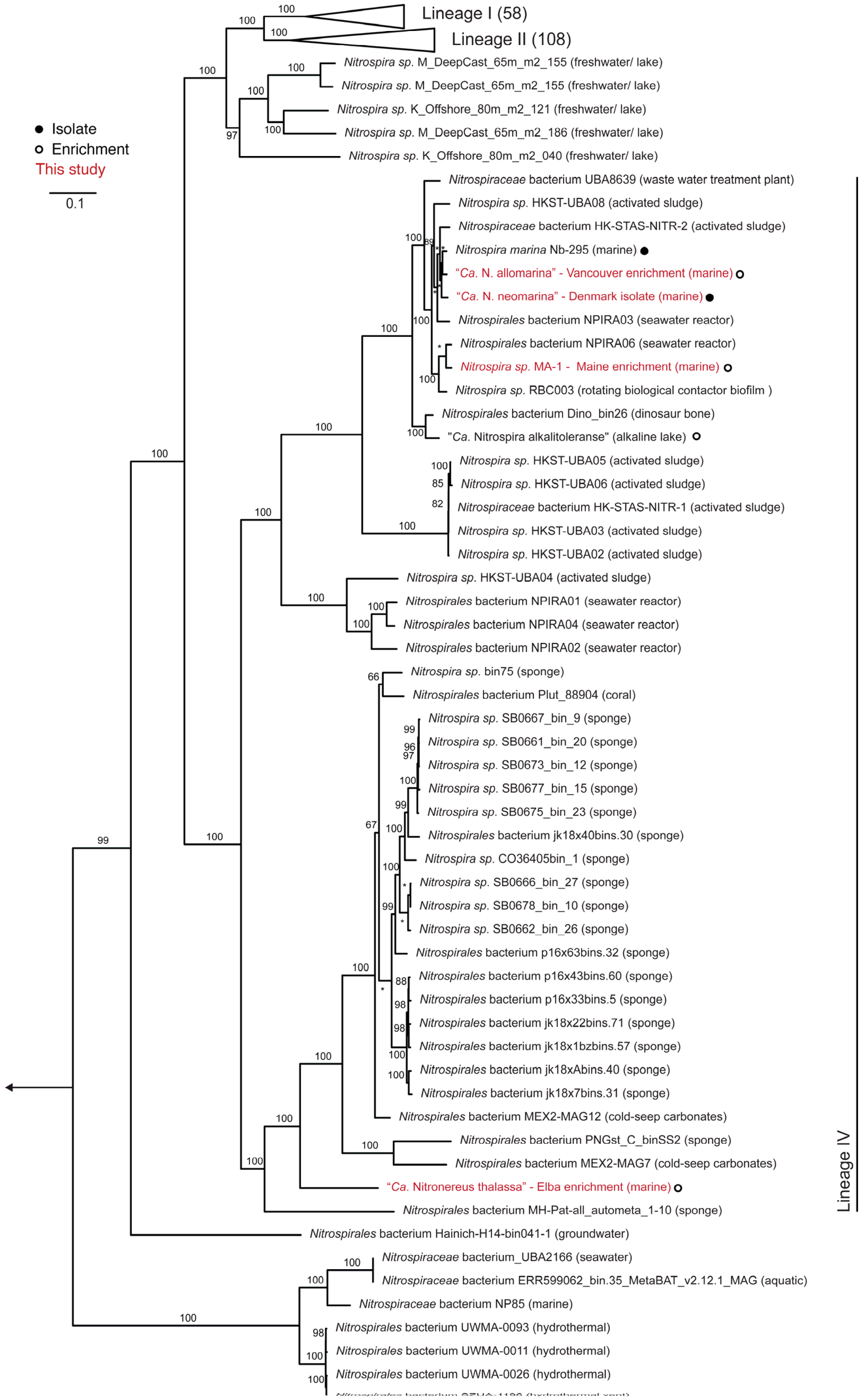
Lineage I (58)
Lineage II (108)
Nitrospira sp. M_DeepCast_65m_m2_155 (freshwater/ lake)
Nitrospira sp. M_DeepCast_65m_m2_155 (freshwater/ lake)
Nitrospira sp. K_Offshore_80m_m2_121 (freshwater/ lake)
Nitrospira sp. M_DeepCast_65m_m2_186 (freshwater/ lake)
Nitrospira sp. K_Offshore_80m_m2_040 (freshwater/ lake)
Isolate
Enrichment
This study
0.1
Nitrospiraceae bacterium UBA8639 (waste water treatment plant)
Nitrospira sp. HKST-UBA08 (activated sludge)
Nitrospiraceae bacterium HK-STAS-NITR-2 (activated sludge)
Nitrospira marina Nb-295 (marine)
"Ca. N. allomarina" - Vancouver enrichment (marine)
"Ca. N. neomarina" - Denmark isolate (marine)
Nitrospirales bacterium NPIRA03 (seawater reactor)
Nitrospirales bacterium NPIRA06 (seawater reactor)
Nitrospira sp. MA-1 - Maine enrichment (marine)
Nitrospira sp. RBC003 (rotating biological contactor biofilm)
Nitrospirales bacterium Dino_bin26 (dinosaur bone)
"Ca. Nitrospira alkalitolerans" (alkaline lake)
Nitrospira sp. HKST-UBA05 (activated sludge)
Nitrospira sp. HKST-UBA06 (activated sludge)
Nitrospiraceae bacterium HK-STAS-NITR-1 (activated sludge)
Nitrospira sp. HKST-UBA03 (activated sludge)
Nitrospira sp. HKST-UBA02 (activated sludge)
Nitrospira sp. HKST-UBA04 (activated sludge)
Nitrospirales bacterium NPIRA01 (seawater reactor)
Nitrospirales bacterium NPIRA04 (seawater reactor)
Nitrospirales bacterium NPIRA02 (seawater reactor)
Nitrospira sp. bin75 (sponge)
Nitrospirales bacterium Plut_88904 (coral)
Nitrospira sp. SB0667_bin_9 (sponge)
Nitrospira sp. SB0661_bin_20 (sponge)
Nitrospira sp. SB0673_bin_12 (sponge)
Nitrospira sp. SB0677_bin_15 (sponge)
Nitrospira sp. SB0675_bin_23 (sponge)
Nitrospirales bacterium jk18x40bins.30 (sponge)
Nitrospira sp. CO36405bin_1 (sponge)
Nitrospira sp. SB0666_bin_27 (sponge)
Nitrospira sp. SB0678_bin_10 (sponge)
Nitrospira sp. SB0662_bin_26 (sponge)
Nitrospirales bacterium p16x63bins.32 (sponge)
Nitrospirales bacterium p16x43bins.60 (sponge)
Nitrospirales bacterium p16x33bins.5 (sponge)
Nitrospirales bacterium jk18x22bins.71 (sponge)
Nitrospirales bacterium jk18x1bzbins.57 (sponge)
Nitrospirales bacterium jk18xAbins.40 (sponge)
Nitrospirales bacterium jk18x7bins.31 (sponge)
Nitrospirales bacterium MEX2-MAG12 (cold-seep carbonates)
Nitrospirales bacterium PNGst_C_binSS2 (sponge)
Nitrospirales bacterium MEX2-MAG7 (cold-seep carbonates)
"Ca. Nitronereus thalassa" - Elba enrichment (marine)
Nitrospirales bacterium MH-Pat-all_autometa_1-10 (sponge)
Lineage IV
Nitrospirales bacterium Hainich-H14-bin041-1 (groundwater)
Nitrospiraceae bacterium_UBA2166 (seawater)
Nitrospiraceae bacterium ERR599062_bin.35_MetaBAT_v2.12.1_MAG (aquatic)
Nitrospiraceae bacterium NP85 (marine)
Nitrospirales bacterium UWMA-0093 (hydrothermal)
Nitrospirales bacterium UWMA-0011 (hydrothermal)
Nitrospirales bacterium UWMA-0026 (hydrothermal)

Figure S3. Phylogeny of the *Nitrospirales* based on 120 conserved proteins. Maximum likelihood tree based on a concatenated alignment of 120 conserved bacterial proteins from representative *Nitrospirales* genomes and MAGs. The newly cultured *Nitrospirales* described in this study are highlighted in red. Isolated or enriched organisms are indicated by full and open circles, respectively. Please refer to Materials and Methods and Table S2 for details of the sequence dataset used to calculate this tree. *Leptospirillum ferrooxidans*, a member of the phylum *Nitrospirota* outside the *Nitrospirales*, was used as outgroup. Numbers on the branches indicate ultrafast bootstrap support (*n*=1000). The lineages I, II, and IV are indicated as described elsewhere (main text refs. 10,30). Sample sources are shown in parentheses. The scale bar shows 0.1 estimated substitutions per amino acid.

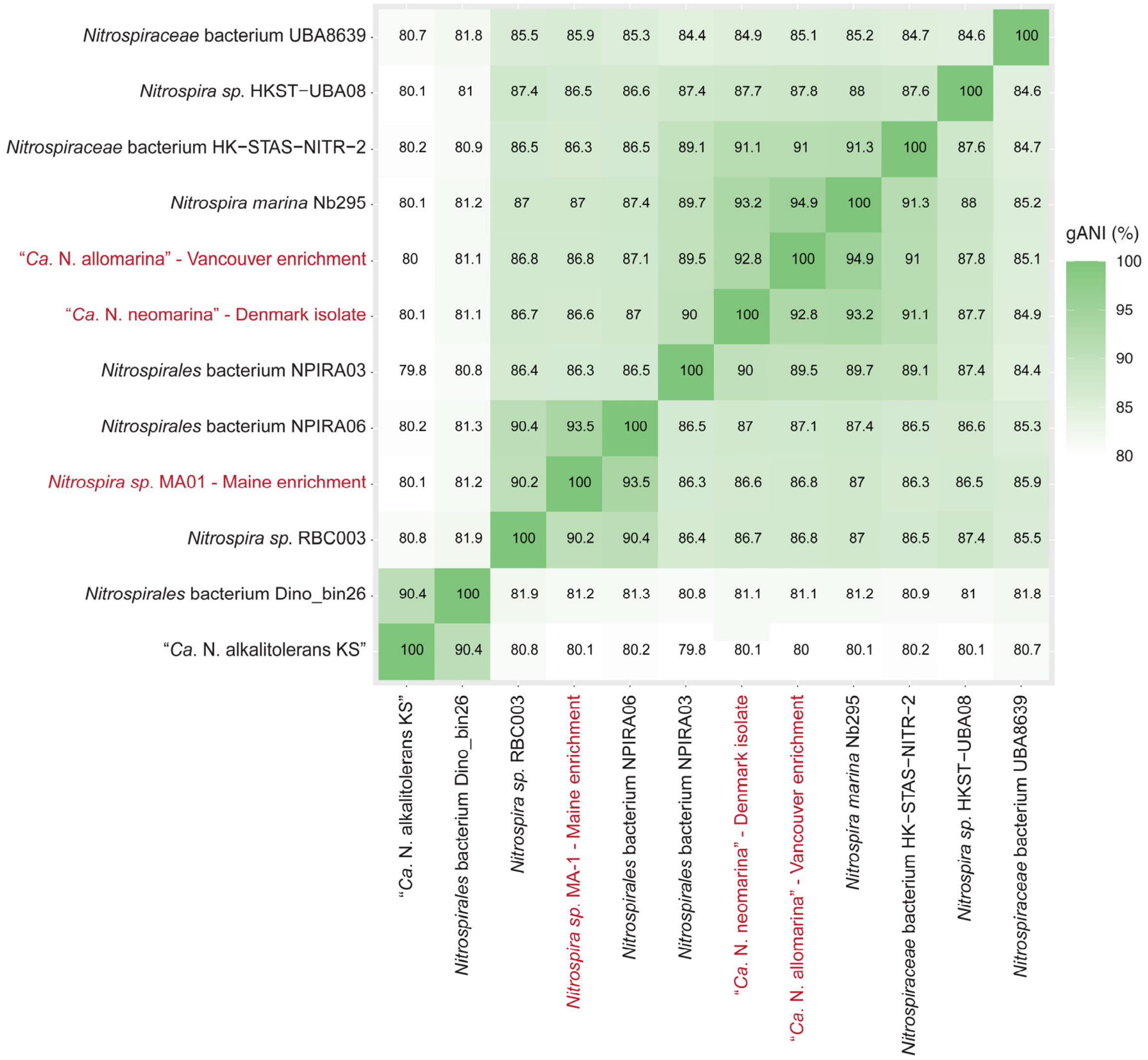

Figure S4. Average genome-wide nucleotide identities of selected lineage IVa *Nitrospira*. Pairwise average whole-genome nucleotide identities (gANI) were calculated for lineage IVa *Nitrospira* that belong to the same genus as *N. marina* Nb-295 according to both our AAI analysis and GTDB (Fig. S5). The organisms are ordered by their phylogenetic placement in the phylogenetic tree in main text Fig. 3. The new *Nitrospirales* described in this study are highlighted in red. Numbers in squares are the gANI values in percent.

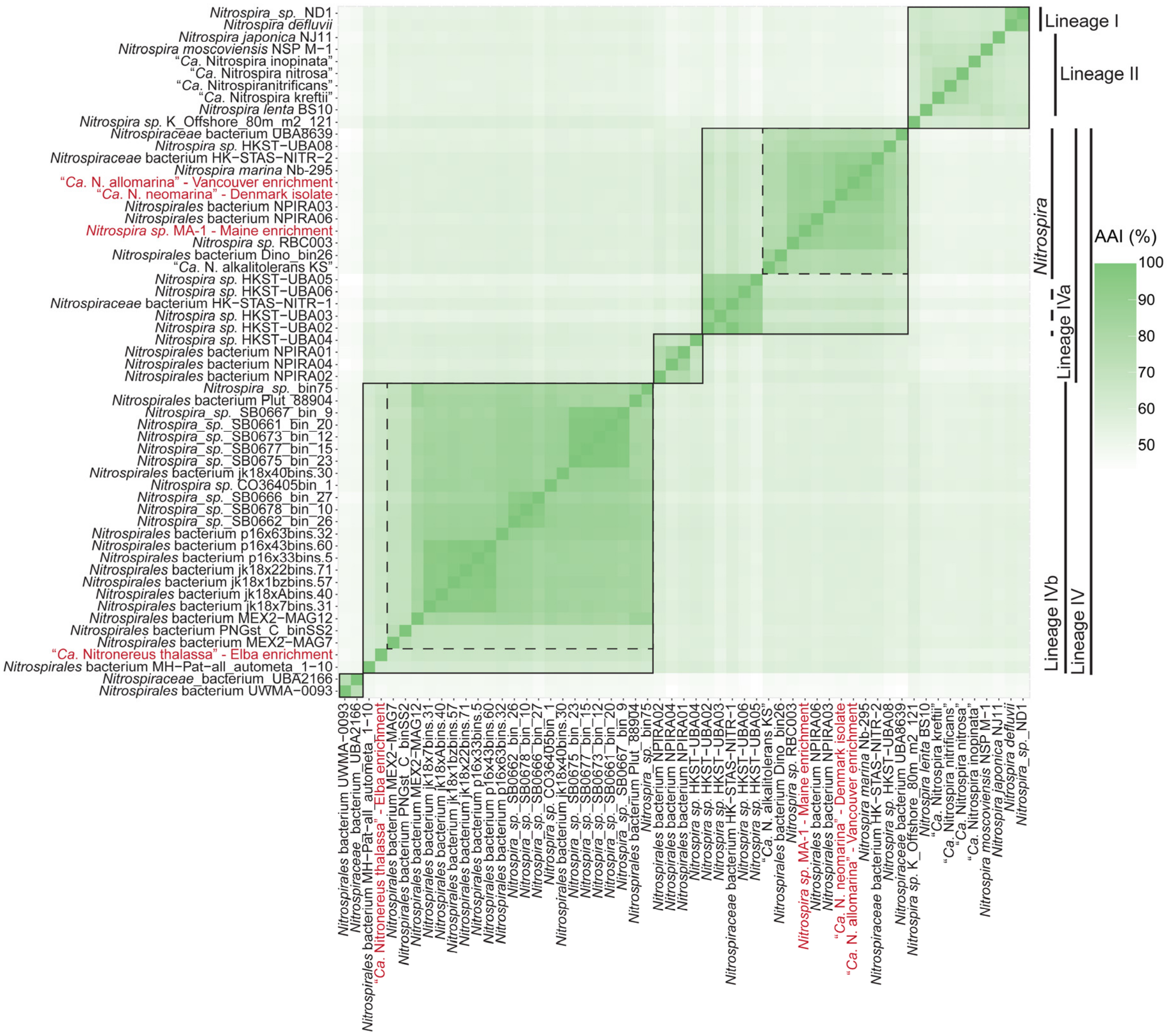

Figure S5. Average amino acid identities of selected *Nitrospirales* members. Pairwise average amino acid identities were calculated for the lineage IV *Nitrospirales* (Fig. 3), selected environmental MAGs, and the cultured terrestrial *Nitrospirales*. The sequences are ordered by their placement in the phylogenetic tree in main text Fig. 3. The new *Nitrospirales* described in this study are highlighted in red. Solid boxes indicate a tentative genus level boundary of 60% amino acid identity. Dashed boxes indicate putative genera based on the GTDB (Table S2) for the lineage IV *Nitrospirales*. The extent of the genus *Nitrospira* based on AAI or GTDB is

indicated by a solid and a dashed line to the right of the figure, respectively. Lineages I, II, V, IVa and IVb are shown by solid lines.

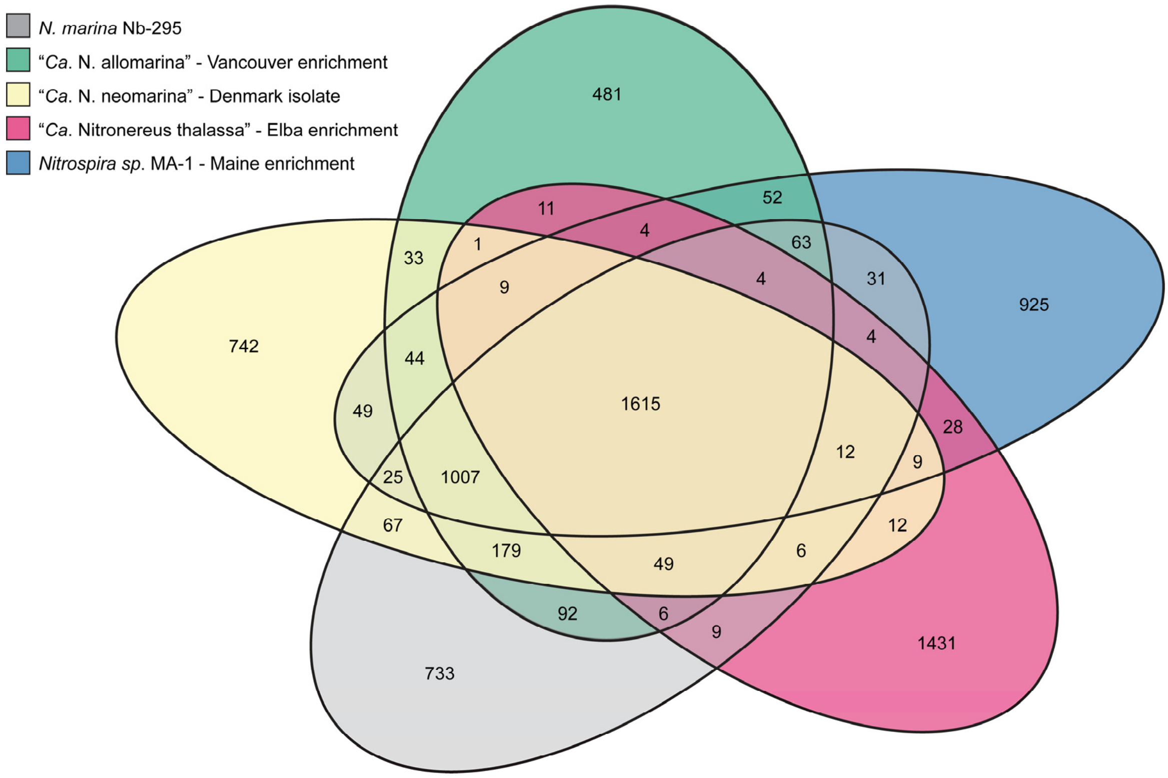

Figure S6. Pan-genome analysis of marine *Nitrospirales*. The Venn diagram depicts a pan-genome analysis of the newly cultured marine *Nitrospirales* from this study and *N. marina* Nb-295. The numbers of shared or unique homologous proteins are indicated. The diagram was generated in MaGe using the MICFAM parameters 50% amino acid identity and 80% alignment coverage (main text ref. 28).

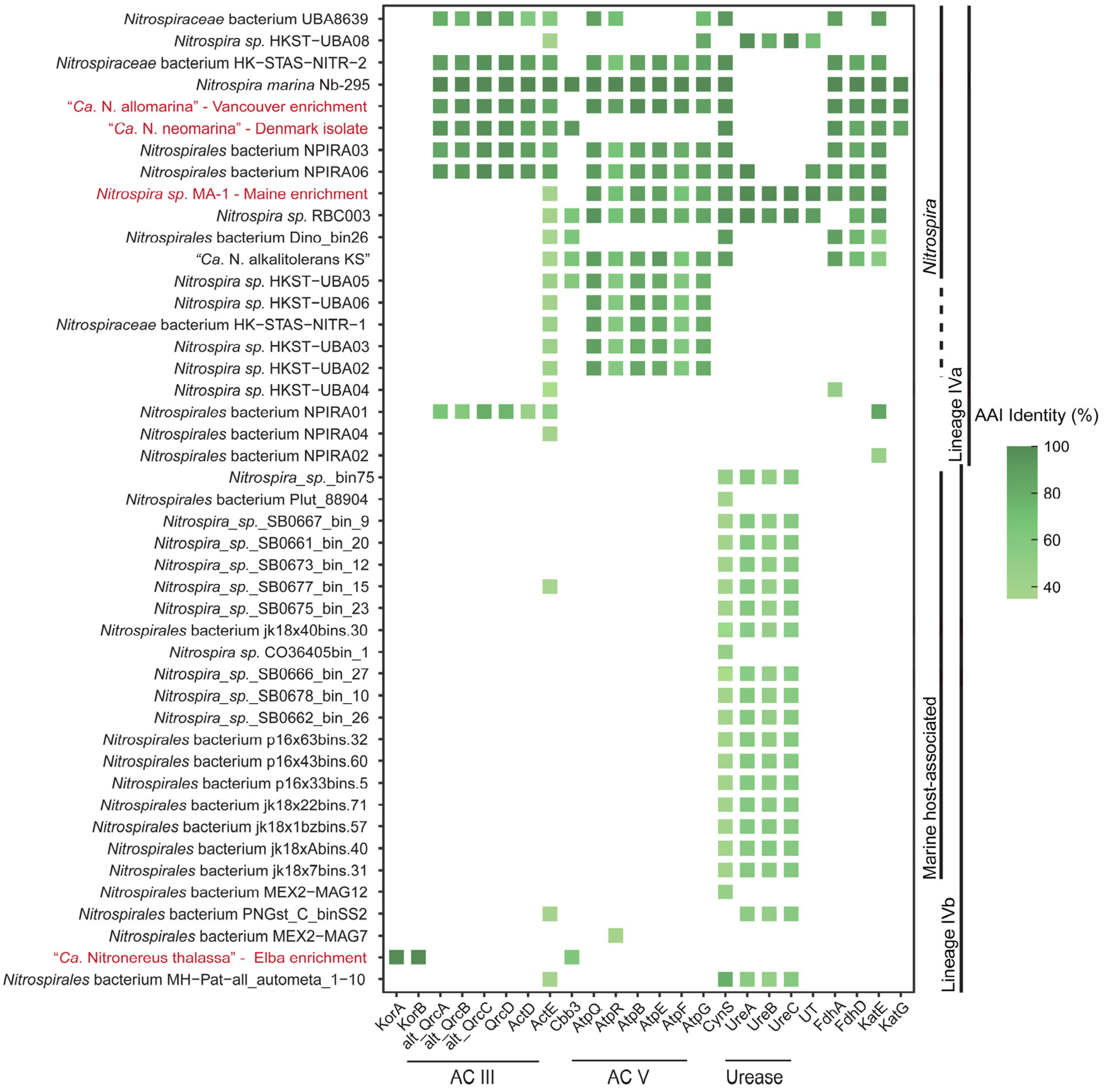

Figure S7. Distribution of selected proteins within the lineage IV *Nitrospirales*. The colored squares depict the presence of these proteins (Table S7) within the *Nitrospirales* lineage IV genomes based on a blastP search (alignment length ≥ 50% and amino acid sequence identity ≥ 35%). Color intensity indicates the percent identity of the aligned amino acid sequences. The lineages are indicated as in Fig. S5. The full names of the proteins are listed in Tables S6 and S7.

UT, urea transporter; AC, alternative complex. Roman numbers indicate respiratory chain complexes.
